# Mathematical Modelling of Polyamine Metabolism in Bloodstream-Form *Trypanosoma brucei*: An Application to Drug Target Identification

**DOI:** 10.1371/journal.pone.0053734

**Published:** 2013-01-23

**Authors:** Xu Gu, David Reid, Desmond J. Higham, David Gilbert

**Affiliations:** 1 Medical Research Council Human Genetics Unit, Medical Research Council Institute of Genetics and Molecular Medicine, University of Edinburgh, Western General Hospital, Edinburgh, United Kingdom; 2 Gold Standard Simulations Ltd., The Rankine Building, Glasgow, United Kingdom; 3 Department of Mathematics and Statistics, University of Strathclyde, Glasgow, United Kingdom; 4 School of Information Systems, Computing and Mathematics, Brunel University, Uxbridge, United Kingdom; University of Westminster, United Kingdom

## Abstract

We present the first computational kinetic model of polyamine metabolism in bloodstream-form *Trypanosoma brucei*, the causative agent of human African trypanosomiasis. We systematically extracted the polyamine pathway from the complete metabolic network while still maintaining the predictive capability of the pathway. The kinetic model is constructed on the basis of information gleaned from the experimental biology literature and defined as a set of ordinary differential equations. We applied Michaelis-Menten kinetics featuring regulatory factors to describe enzymatic activities that are well defined. Uncharacterised enzyme kinetics were approximated and justified with available physiological properties of the system. Optimisation-based dynamic simulations were performed to train the model with experimental data and inconsistent predictions prompted an iterative procedure of model refinement. Good agreement between simulation results and measured data reported in various experimental conditions shows that the model has good applicability in spite of there being gaps in the required data. With this kinetic model, the relative importance of the individual pathway enzymes was assessed. We observed that, at low-to-moderate levels of inhibition, enzymes catalysing reactions of *de novo* AdoMet (MAT) and ornithine production (OrnPt) have more efficient inhibitory effect on total trypanothione content in comparison to other enzymes in the pathway. In our model, prozyme and TSHSyn (the production catalyst of total trypanothione) were also found to exhibit potent control on total trypanothione content but only when they were strongly inhibited. Different chemotherapeutic strategies against *T. brucei* were investigated using this model and interruption of polyamine synthesis via joint inhibition of MAT or OrnPt together with other polyamine enzymes was identified as an optimal therapeutic strategy.

## Introduction

The development of drugs to combat human African trypanosomiasis (HAT) has become a major public concern due to toxicity, inefficacy and availability problems with current drug treatments [1], [2]. Identification of potential drug targets within the *T. brucei* parasite is an invaluable tool for designing chemotherapeutic agents against the disease. A challenge in drug design arises from the similarity of metabolic pathways in parasitic protozoa and their mammalian hosts, resulting in toxicity to the host as well as the parasite. Anti-parasitic drugs that are efficient, non-toxic and affordable are urgently required.

Polyamines are ubiquitous cellular components that are essential for cell growth and division. Polyamine metabolism in mammalian cells has previously been studied using mathematical modelling [3]. Polyamine metabolism in *T. brucei* has a number of key features that distinguish it from polyamine metabolism in mammals. The major differences lie in the specificity of metabolites and enzymes as well as the associated regulation patterns. Most notably, the enzyme s-adenosylmethionine decarboxylase (AdoMetDC) is activated through dimerisation with an enzymatically inactive homologue termed prozyme. Moreover, spermidine (Spd), in addition to its plethora of other cellular roles (e.g. serving as an important inducer for the compact form of DNA), in trypanosomatids, is linked to two molecules of glutathione to yield the redox active metabolite trypanothione, 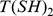, which is a compound critical for trypanosome viability and virulence.

Trypanosomes are sensitive to inhibition of the polyamine pathway. For example, it has been shown that trypanosomes depend on Spd for growth and survival, which ceases when the level of Spd drops below a certain threshold [4]. There is therefore considerable therapeutic potential in compounds that disrupt polyamine biosynthesis. The suicide inhibitor eflornithine (difluoromethylornithine, DFMO) kills trypanosomes by irreversibly interacting with ornithine decarboxylase (ODC) leading to diminished polyamine levels. DFMO is now the first line treatment used in HAT therapy. Inhibitors of AdoMetDC [5] have also been shown to be potently trypanocidal. These features have ensured that the polyamine pathway in *T. brucei* has been subject to investigation and details are available for enough of the enzymes to allow a mathematical model to be constructed. A recent attempt to model trypanothione (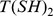) metabolism in *Trypanosoma cruzi* (*T. cruzi*) [6] also points to the value in modelling of this branch of metabolism in trypanosomatids.

Dynamic behaviour of complex biological systems is not deduced easily from collective descriptions of its individual parts, requiring instead a systematic approach with advanced computational technology. Mathematical modelling offers a route to achieve a system-level understanding [7], [8]. In the context of biological systems, mathematical models of metabolism allow improved understanding of the contribution of individual enzymes to the larger system. This can be achieved by studying the rates at which system components interact and physical laws that govern the reactions. Good models enable interpretation and predictions about the consequences of pathway perturbation that can supplement or even replace *in vivo* or *in vitro* experiments. Without a reliable model, it is difficult to elucidate how complex properties of dynamic systems arise from nonlinear enzymatic interactions.

In this paper, we develop the first kinetic model of polyamine metabolism in blood-stream form *T. brucei*, derived from published information related to system components and their interactions. We are interested in seeking a model to reproduce what has already been observed and also to make predictions about the system to suggest future experiments and guide drug design. Since mathematical models are manipulable, the mechanisms underlying the metabolic regulation of polyamine biosynthesis can be evaluated *in silico*. This kinetic model aims at understanding the effectiveness of the anti-trypanosomal drug DFMO in detail and examining other polyamine enzymes as potential targets for anti-trypanosomal chemotherapy.

## Results

As this is the first model of polyamine metabolism in *T. brucei*, we shall summarise the main points from the the model design procedure before presenting the simulation results of the kinetic model. More details on the construction of the model are given under Materials and Methods.

**Figure 1:**
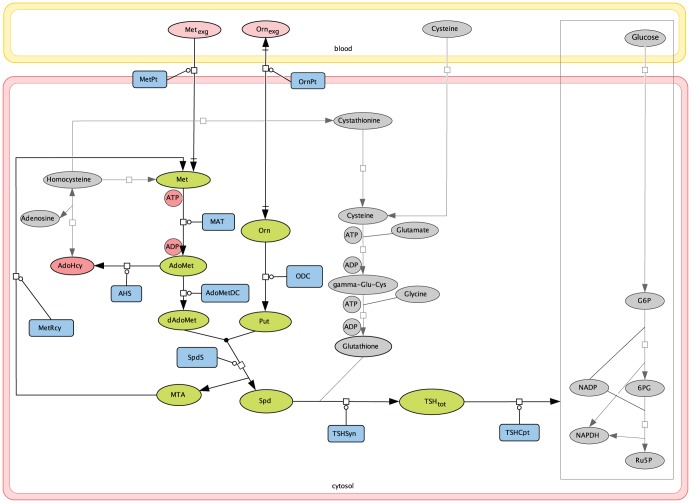
A detailed graphical representation of total trypanothione metabolism. Edges represent chemical conversions between model components with arrows indicating reaction directionality. Metabolites and reactions constituting the polyamine biosynthetic pathway that are considered in this model are emphasised with bold type, with time-variant metabolites shown in green and constant metabolites shown in pink. Enzymes catalysing each active elementary step in the pathway are denoted with blue boxes. The remaining modules of the network shown in grey are not modelled but help gaining an overall picture of the metabolism. Abbreviations of polyamine metabolites: Met, methionine; AdoMet, S-adenosylmethionine; dAdoMet, decarboxylated AdoMet; MTA, methylthioadenosine; AdoHcy, S-adenosylhomocysteine; Orn, ornithine; Put, putrescine; Spd, spermidine; 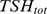, total trypanothione; 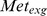, exogenous methionine; 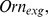 exogenous ornithine. Abbreviations of intra-cellular polyamine enzymes: MetPt, Met uptake enzyme; MAT, AdoMet synthase; AHS, methyltransferase; AdoMetDC, AdoMet decarboxylase; MetRcy, Met recycling enzyme; OrnPt, Orn uptake enzyme; ODC, Orn decarboxylase; SpdS, Spd synthase; TSHSyn, 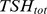 synthesis catalyst; TSHCpt, 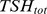 consumption catalyst.

A detailed schematic representation of the trypanothione metabolic network is depicted in Figure 1. This diagram indicates the complex interconnections between the main pathways, composed in parallel, which comprise the network. These are the polyamine biosynthetic pathway for the production of Spd, the glutathione biosynthetic pathway for the production of glutathione and pentose phosphate pathway for the production of NADPH mediating the reduced trypanothione redox cycle from oxidised trypanothione disulfide. Spermine, which is a critical polyamine in mammalian cells, is not taken into account due to its negligible role in *T. brucei* [9]–[12]. Here we study the contribution of the polyamine biosynthetic pathway to regulation of the total trypanothione contents (the summation of both reduced and oxidised trypanothione, 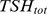 for short). In *T. brucei* the lack of a classical arginase [13] has led to the identification of ornithine (Orn) uptake from blood as the main mechanism to accumulate this metabolite, serving as the only source for intracellular Orn in our model. Metabolites and enzymes constituting the polyamine pathway are emphasised with bold type in Figure 1.

Model development involved converting the reaction scheme of interest in Figure 1 into a set of ordinary differential equations (ODEs). In our model the polyamine biosynthetic pathway is described mathematically by eight ODEs, which associate the changes in concentration levels of system components with the rate equations of enzymatic reactions involved. Some practical considerations had to be taken into account when designing the structure of the model in order to study this pathway in isolation from the entire network.

Michaelis-Menten kinetics (for one substrate) has been used to model enzymatic velocities of the MTA recycling enzyme (MetRcy) and the transporter of exogenous Met (MetPt). Michaelis-Menten kinetics with two substrates (rapid equilibrium random bi-bi mechanisms) has been applied for the enzymes SpdS and MAT. More complex mechanisms have been employed for ODC, exogenous Orn uptake (OrnPt), AdoMetDC, TSHSyn and TSHCpt to which standard Michaelis-Menten kinetics are not sufficient to explain their behaviour. By comparing experimental data with model predictions, we iteratively refined the mathematical representations of enzyme kinetics to render the model satisfactory.

The incomplete knowledge of parameter values makes parameter estimation a necessary step prior to dynamic simulations. In our study, simultaneous fitting against both the physiological steady state and *in vivo* DFMO-mediated polyamine inhibition reported by Fairlamb et al. [11] was applied to tune the unknown parameters of the given model structure. DFMO-induced perturbation is the most comprehensive data source available for training the model (inhibition profiles being given for 6 out 8 metabolites of the pathway in *T. brucei*). Gene perturbation measurements on ODC [14], SpdS [14], [15], prozyme [16], AdoMetDC [16], [17] and trypanothione synthetase [18], which were not used for training the model, are then employed as validation data to evaluate the given model structure.

It is important to point out that this modelling activity is not only challenged by the lack of prior knowledge, i.e. several kinetic parameters are absent, but also by the fact that experimental observations involve different trypanosome strains grown in different conditions - work by Fairlamb et al. was from trypanosomes grown in rats whilst other gene-perturbation experiments involved in vitro cultivated strains. Inevitably, therefore, absolute quantification of metabolite levels which is strain and growth condition sensitive cannot emerge from such limited studies, although the general trends in quantification are conserved.

### Model training

The ‘best’ set of parameter estimates from simultaneous fitting against both the steady-state and DFMO-perturbed profiles is reported in Text S1. Details on parameter estimation are given in the Model Calibration section of Materials and Methods. Model metabolites simulated with the ‘best’ set of parameter estimates reached a steady state after less than the simulation time period of two days and maintained it until the end of day 6. A good match between steady-state levels of polyamine metabolites from model predictions (termed the *basal* condition) and the reference data is shown in Table 1. We further investigated model sensitivity to different initial concentrations of pathway metabolites (varied by up to 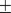
80% of the estimated initial values reported in Table S1). We found that the behaviour of these model variants converged to almost the same basal condition over a simulated time span of 4 days, indicating good stability (see Figure S1).

**Table 1:** Basal condition of polyamine concentrations.

	Met	AdoMet	dAdoMet	Orn	Put	MTA	Spd	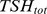
**from Model (** 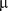 **M)**	3341.5	20.3	26.2	86.2	180.7	20	2049	340
**from refs. (** 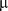 **M) ** [**11**]	3978	19	9	43	517	20	2069	340

**Figure 2:**
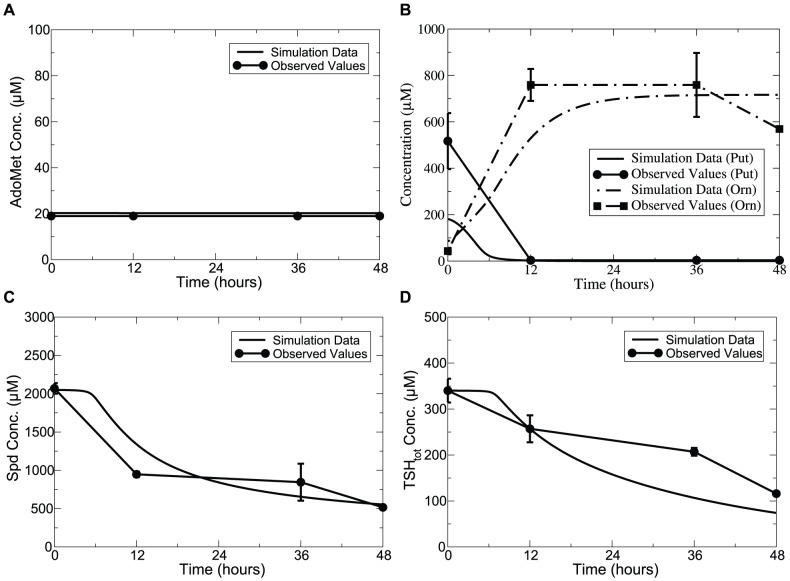
Time-series simulation of DFMO effects on polyamine levels compared with experimental data. *Lines without symbols*, model predictions; *lines with symbols*, experimental observations from [11]. The maximum velocity of ODC was modelled as a time-dependent variable, with the activity decreased by more than 99% within 12-hour of treatment with DFMO. AdoMet dynamics observed by Xiao et al. [14] were adopted. Error bars are presented where appropriate data was available in the original papers.

Model simulations of DMFO induction over an interval of 48 hours show good agreement with experimental data in terms of both exact values and transient changes in the metabolite concentrations, as shown in Figure 2. A drastic decrease of Put was captured accompanied by a decrease in Spd. AdoMet was well fitted, and remained unchanged as observed in [14]. This may be attributed to the fact that free-form AdoMetDC is insensitive to the reaction product dAdoMet as indicated by the high value of 970.6 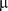
M predicted for the product inhibition parameter, which agrees with the hypothesis made in [19]. An increase of Orn was observed within the first 12 hours of DFMO treatment, followed by attainment of an accurate steady state. Dynamics of 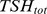 was also well captured compared with the measurements reported for the reduced trypanothione. Note that when plotting the time course of polyamines under perturbed conditions, the *basal* condition acts as the initial status for the simulation of DFMO-treated model, which also applies to model simulation under other perturbed conditions investigated below.

### Model validation

Comparison between model predictions using estimated parameter values and independent data sets obtained from distinct states of the system allows assessment of model use. To this end, data from available drug treatment and gene-knockdown perturbation experiments on ODC [14], SpdS [14], [15], prozyme [16], AdoMetDC [16], [17] and trypanothione synthetase [18] are used as validation data. When simulating the model for each of the perturbation experiments, the wild-type value of the maximum velocity for each individual enzyme (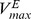 with *E* representing the specific enzyme name) is replaced with an exponential decay function of the form of 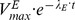 in the corresponding rate equations, which aims to mimic the inhibition of individual enzymes over time (*t*). An exponential decay constant, 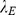, was derived for individual instances by parameter fitting according to the given inhibitory profiles of corresponding enzymes. For all other kinetic parameters, the values were fixed at those reported in Text S1.

### Model predictions on the consequences of ODC knockdown

**Figure 3:**
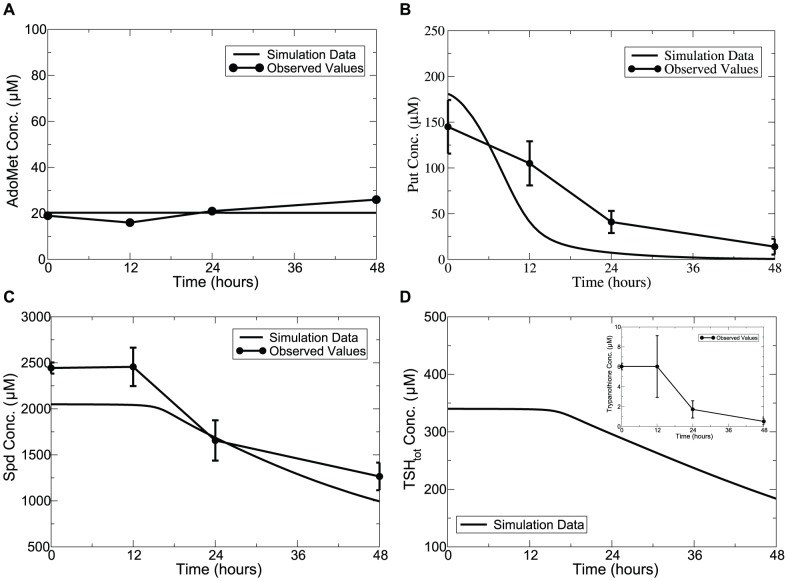
Time-series simulation of ODC inhibition on polyamine levels compared with observed values. *Lines without symbols*, model predictions; *lines with symbols*, experimental observations from [14]. The maximum velocity of ODC was modelled as a time-dependent variable during the simulation with 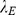 equal to 0.0016, where the ODC activity was decreased by 90% within 24 hours of RNAi induction. Error bars are presented where appropriate data was available in the original papers.

DFMO is used to treat HAT and acts by inhibiting ODC with knock on effects on polyamine production, for example, reducing Put and Spd. As shown in Figure 3, our model replicated the reduction in concentrations of Put, Spd and 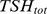 over 48 hours of model simulation where ODC activity is reduced by 90% within 24 hours of induction (as specified in [14]). dAdoMet serves to provide the aminopropyl group in Spd production which accumulates dramatically, while AdoMet is unchanged as reported in [14].

### Model predictions on the consequences of SpdS knockdown

**Figure 4:**
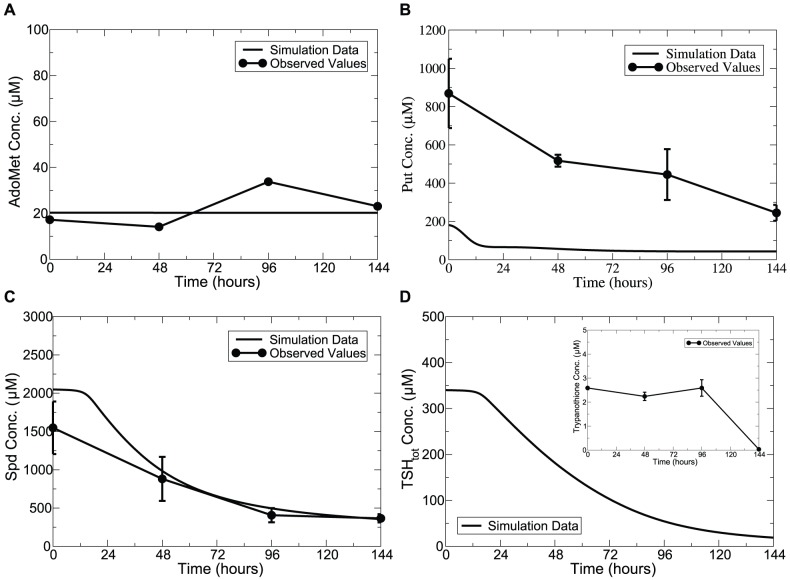
Time-series simulation of SpdS inhibition on polyamine levels compared with observed values. *Lines without symbols*, model predictions; *lines with symbols*, experimental observations from [14]. The maximum velocity of SpdS was modelled as a time-dependent variable with 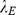 equal to 0.0016. Error bars are presented where appropriate data was available in the original papers.

Spd plays multiple roles in trypanosomes including a critical role in producing the redox reactive thiol metabolite trypanothione (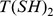), which underlines the sensitivity of trypanosomes to the loss of Spd through reduced capability to maintain cellular redox. SpdS has been validated as a potential drug target in *T. brucei* [14], [15]. Xiao et al. [14] observed that after 6 days of RNAi-mediated Spd depletion (SpdS activity knocked down by 90% within 2 days of induction), Spd and 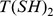 decreased to 20% and 5% of the uninduced controls. Our model predicted a similar trend in changes in concentrations, namely that Spd and 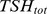 are reduced to 17% and 6% of the controls, as shown in Figure 4C and Figure 4D. No significant changes were found for AdoMet and our model predicted this as well for this metabolite (see Figure 4A).

Put is an interesting metabolite regarding its response to SpdS down-regulation. Xiao et al. reported a 45% decrease in Put concentration over 3 days after SpdS depression. Taylor et al. [15] also showed that, within 3 days, repressing SpdS to just 5% compared to wild type caused a 60% decline in Spd contents but, unexpectedly, no significant build up of Put was found. In *T. brucei* therefore, cellular overproduction of Put is avoided, possibly as excessive Put can elicit oxidative stress as reported in mammalian cells [20], [21].

One refinement we made during the model building procedure was to introduce a term reflecting the plausible regulation of SpdS on ODC activity (defined in Equation 1 in the Model Descriptions section of Materials and Methods) which serves to prevent excessive Put accumulation in the case of SpdS perturbation, as demonstrated in Figure 4B. We observed that when this term is removed from the model while keeping the remaining parameters unchanged, a 90% knockdown of SpdS leads to a dramatic buildup in Put level (see Text S4). Inclusion of this regulatory term enables the model to simulate experimental observations. It will now be of interest to determine the biological basis of this regulation.

### Model predictions on the consequences of AdoMetDC knockdown and prozyme knockout

**Figure 5:**
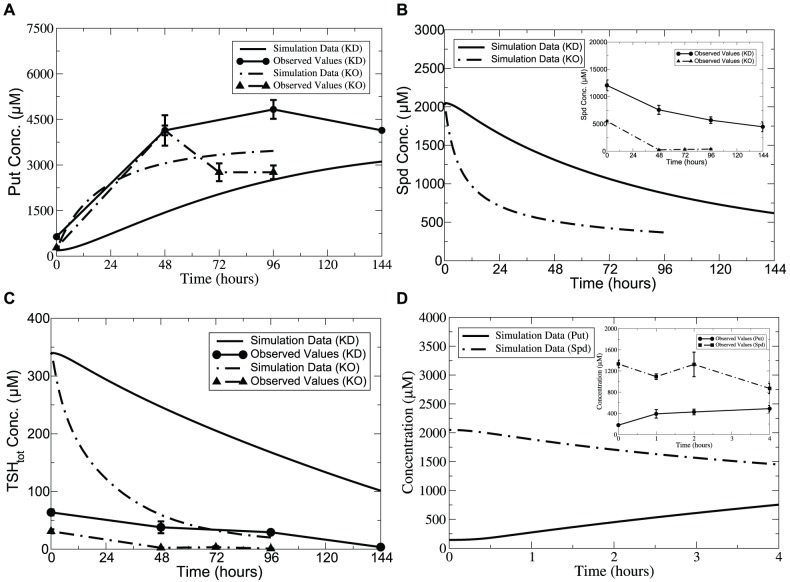
Time-series simulation of AdoMetDC inhibition on polyamine levels compared with observed values. *Lines without symbols*, model predictions; *lines with symbols*, experimental observations from [16] for (A) to (C) and [17] for (D). In (A) to (C), during knockdown (KD) simulations, total AdoMetDC concentration ([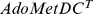]) was modelled as a time-dependent variable with 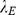 equal to 0.0004 to represent the 70% activity down-regulation within 2 days of induction; during knockout (KO) simulations, the factor 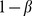 representing the percent of the complex AdoMetDC|prozyme taking up the total enzyme AdoMetDC is set to zero to represent full prozyme removal. In (D), MDL effects on Put and Spd dynamics were plotted. During the simulation, total enzyme concentration of AdoMetDC was modelled using a exponential decay function with 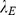 set to 0.07 to mimic a 98% knockdown within 1 hour of induction as specified experimentally. Error bars are presented where appropriate data was available in the original papers.

AdoMetDC has already been validated as a drug target in *T. brucei*. Loss of AdoMetDC or prozyme was observed to lead to decreases in Spd and 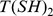 and to cell death [16]. In our model, simulations of prozyme knockout (over a simulated time span of 4 days with a complete removal of the ligand-binding form of AdoMetDC) and AdoMetDC knockdown (over a simulated time span of 6 days with a 70% down-regulation of total AdoMetDC concentration within 2 days of induction, as specified in [16]) both resulted in a large increase in Put levels and substantial reduction in Spd and 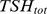. Simulation of the time-dependent effects on polyamine levels of Put, Spd and 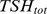, induced by AdoMetDC knockdown and complete prozyme knockout are reported in Figure 5A–5C. An 80% reduction due to prozyme knockout versus 65% reduction from AdoMetDC knockdown for Spd and a 94% reduction due to prozyme knockout versus 70% reduction from AdoMetDC knockdown for 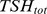, were seen. These results are in good agreement with the tendencies described by real experimental observations [16].

We further compared the resulting 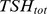 content when the same degree of inhibition (70% knockdown applied to total AdoMetDC concentration) was applied to ODC. Our model predicted a relatively lower 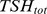 level at the end of the simulated time span of 4 days from AdoMetDC inhibition (70% depletion) compared with that from ODC inhibition (40% depletion), which agrees with [17] that AdoMetDC could be a more promising chemotherapeutic target than ODC for *T. brucei*. Additionally, a 70% AdoMetDC knockdown or prozyme knockout caused an almost full depletion of dAdoMet accompanied by a 6-fold increase in Orn while AdoMet remained constant. These model predictions can be verified when the relevant experimental data is available. Our model simulations also reveal that activity of free-form (homodimeric) AdoMetDC (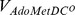) is 0.03% of the activity of heterodimer AdoMetDC
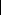
prozyme (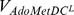), which is consistent with the experimental observations [16] that the former is as low as 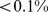 of the latter, indicating that prozyme reacting with AdoMetDC is a limiting factor for AdoMetDC activity.

Our model has also been validated on the consequences of inhibiting AdoMetDC activity by a specific inhibitor MDL73811 (5′- {[(Z)-4-amino-2-butenyl]methylamino}-5′-deoxyadenosine). When AdoMetDC activity was almost completely inhibited (to 2% of control value within 1 hour of administration), a modest 33% decrease in Spd was observed by 4 hours post-administration of MDL73811 [17]. Our model predicted a similar 30% reduction in Spd over a simulated time span of 4 hours in response to the strong AdoMetDC down-regulation (via reducing total AdoMetDC enzyme concentration 
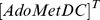
 to 2% of the control value) and a 20% depletion in 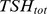 was predicted. Simulation results are depicted in Figure 5D.

One other refinement we made during the model construction procedure was to present the ODC-catalysed reaction with reversible kinetics. This implementation led Put to plateau in response to perturbations of AdoMetDC and prozyme. However, in the case of SpdS perturbation, simply modelling the ODC-catalysed reaction reversibly (without the addition of the postulated regulation between SpdS and ODC) indeed helped to alleviate excessive accumulation of Put, but a 20-fold increase in Put contents was still observed over a simulated time span of 6 days, indicating that the additional regulation of SpdS on ODC is essential regardless in this case. Refer to Text S4 for more details.

### Model predictions on the consequences of TSHSyn knockdown

**Figure 6:**
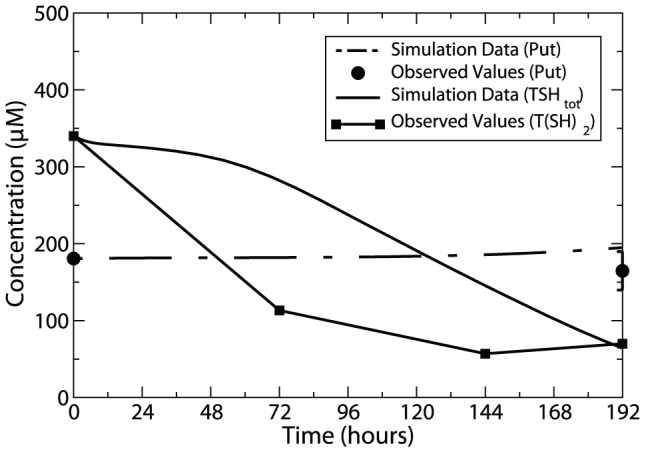
Time-series simulation of TSHSyn inhibition on 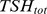 level compared with observed values. *Lines without symbols*, model predictions; *lines with symbols*, experimental observations from [18]. During the simulation, the maximum velocity of TSHSyn was modelled as a time-dependent variable using the exponential decay function with 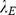 set to 0.00045. Percentage changes of Put and 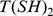 at discrete time points over a simulated time span of 8 days were extracted from [19] and normalised to the basal conditions of respective metabolites. For Put, only the percentage change at the end of the simulated time span was shown, since percentage changes for this metabolite over other time points were not reported in [19].

Trypanothione synthase (TryS), which catalyses production of the reduced trypanothione, 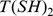, from Spd and glutathione has been recognised as a good drug target for trypansomes [18]. It has been the focus of anti-trypanosomal research, owing not only to its significant role in trypanosomal viability but also its capability in regulating the levels of polyamines, glutathione and glutathione-spermidine conjugates. In our model, this enzyme is represented as TSHSyn and a one-step production of total trypanothione from Spd is assumed (as stated in the Model Descriptions section of Materials and Methods). Ariyanayagam et al. [18] reported that, within 3 days of TryS inhibition, TryS activity decreased 10-fold, giving rise to a 85% reduction in 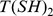 at the end of 8 days of RNAi induction, whereas the reactants of the reaction, Put and Spd, are not significantly increased. Despite the absence of glutathione in the model, knockdown simulations of TSHSyn (following the reported inhibitory profile of TryS) predicted a good match with the measured concentration changes of Put (no profound changes predicted) and 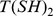 (a 80% decrease in total trypanothione, 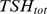, predicted) at the end of simulation duration of 8 days (illustrated in Figure 6).

However, our model predicted a 10-fold increase in Spd level, which contradicts the measured dynamics. We postulate that this may result from the exclusion of glutathione in the model, which is found to accumulate markedly over 8 days of TryS inhibition in our simulation study. A potential elevation in Spd levels could be averted if it reacts with increased glutathione levels to produce 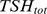. In the absence of quantitative inclusion of glutathione in our model, Spd was unconstrained to be rapidly increased. We tested this hypothesis by combining the above TSHSyn inhibition with an increased utilisation of Spd (modelled through reduction in Spd production rate to 5% of the uncontrolled level at the end of 3 days). The model predicted a 30% drop in Spd accompanied with a considerable (90%) reduction in 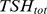, supporting the possibility that Spd levels may be regulated by the interaction with glutathione. When adequate kinetic information becomes available regarding glutathione kinetics and intermediate metabolites in *T. brucei*, integration of the polyamine model with glutathione biosynthesis would be useful for improving quantitative predictions on inhibition consequences.

We also examined the consequences of knockdown of TSHCpt (catalysing the sink reaction of total trypanothione, 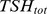) and found that inhibition of TSHCpt increased the concentration level of total trypanothione (shown in Figure S2) but no dramatic changes on other metabolites of the pathway were seen.

## Discussion

### Sensitivity analysis

**Figure 7:**
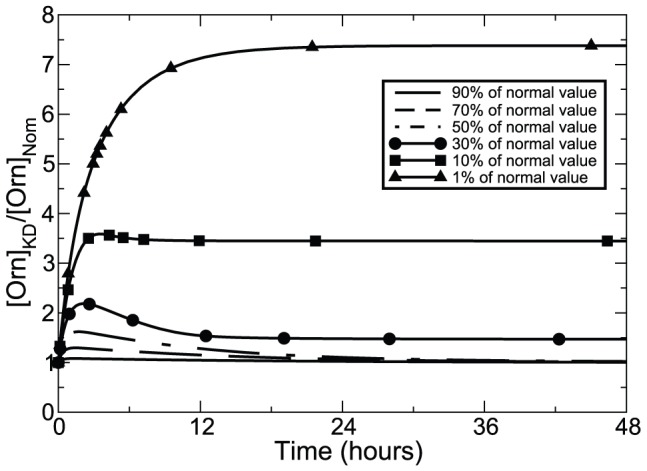
Orn dynamics over 2 days after ODC activity depression. During the simulation, the maximum velocity of ODC was modelled as a time-independent constant by multiplying the normal value by the percentage amount.

Sensitivity analysis describes changes of metabolite concentrations as result of changes in model parameters. We examined model sensitivity properties by running the model with the maximum velocity (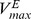) of key pathway enzymes varied independently by 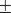
10% of the nominal values. The model then evolves to a new steady state over a simulated time span of 6 days. Changes of maximum activities of enzyme MAT and MetPt resulted in a global effect on the system, whereas some parameters influenced specific metabolites; for example, changes of TSHSyn led specifically to changes of Spd and 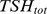 and the function of TSHCpt is limited to 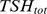 only. The other input to the model, OrnPt, also showed an impact on Orn, Put and 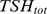. With this analysis, we observed that when ODC is inhibited, Orn built up rapidly over 2 days leading to a new steady state, which is proportional to the degree of knockdown applied to ODC (illustrated in Figure 7). This figure may explain why reversible inhibitors of ODC are not successful in killing trypanosomes as the extensive increase in Orn concentration (almost 7.5 times of the normal Orn value) will out-compete the reversible inhibitors interacting with ODC. The binding of the enzyme with irreversible inhibitors can however prevent competition from the substrate, but the inhibitors have to be sufficiently potent to cause apparent loss of 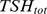 content.

**Figure 8:**
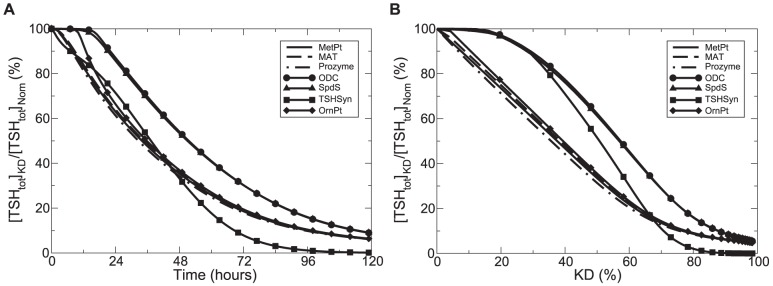
Studies of changes in 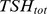 concentration under different perturbation scenarios. In (A) time-series 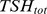 concentration values are calculated over a simulated time span of 5 days subject to a 90% decrease in individual enzyme velocities. A 90% knockdown of AdoMetDC enzyme concentration and a 90% prozyme knockdown were found to follow a similar pattern of 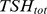 dynamics, and only prozyme inhibition is shown. In (B) 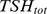 concentration values at the end of the simulated time span (5 days) are calculated subject to various degrees of knockdown (KD) for individual enzymes. In both figures, the percentage of 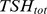 concentration under perturbed (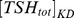) and normal (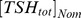) conditions is plotted. In all cases, the maximum velocity of each enzyme is a time-dependent variable subject to specific inhibition within 24 hours of simulation.

We compared the changes in 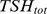 dynamics over a simulated time span of 5 days. Individual enzymes were subject to a 90% knockdown within 24 hours of simulation. These enzymes included ODC, SpdS, prozyme, MAT, OrnPt, MetPt and TSHSyn - enzymes involved in *de novo* synthesis of total trypanothione. Figure 8A indicates that a 90% knockdown of each enzymes led to decreased 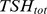, with levels dropped to less than 10% of the unperturbed level at the end of simulation span. MetPt, MAT, prozyme and OrnPt exhibit a much stronger inhibitory effect on 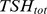 than ODC and SpdS. TSHSyn displayed a faster converging trajectory after 48 hours of simulation and a more complete depletion of 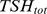 than all other enzymes.

We further analysed 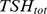 concentration changes (at the end of a simulated time span of 5 days) with respect to different knockdown levels for individual enzymes. Figure 8B indicates that when activity knockdown is more than 70%, TSHSyn has the strongest inhibitory effect on 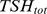, whereas when the knockdown is less than 70%, MAT, MetPt, Prozyme and OrnPt exert the most effective control on 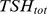 reduction. Under all scenarios, ODC and SpdS displayed a relatively weaker inhibitory impact on 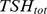. We observed that a 70% loss of ODC and SpdS led to the same effect as a 60% loss of TSHSyn or a 50% reduction of MAT, MetPt, Prozyme or OrnPt, indicating that to achieve the same level of 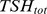 depletion (70%), the knockdown strength required for different enzymes should follow 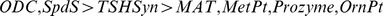. This could point to the enzymes MAT, MetPt, Prozyme and OrnPt as good potential drug targets, which result in the depletion of 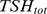 with only small perturbations.

### Combination chemotherapy for *T. brucei*

Enzymes responsible for polyamine biosynthesis are proven drug targets. Simulations generated by our model indicate that strong down-regulation of individual enzymes including ODC, prozyme, SpdS and TSHSyn lead to reductions in 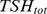 levels, demonstrated to be potential targets for drug design.

The use of mathematical models not only provides a mechanistic understanding but can also drive new and more effective experiments. Combination chemotherapy for African sleeping sickness is attractive as it offers the potential for lower doses of drugs and reduced risk of resistance emerging for individual compounds. The additional requirements for regulatory approval of combination therapies however makes *de novo* production of combination therapies difficult, but it is worth noting that for HAT it was possible to introduce a DFMO-nifurtimox combination therapy (NECT) which has advantages over DFMO monotherapy alone [22], [23]. Metabolomics analysis did not indicate a role in polyamine pathway inhibition by nifurtimox [13], however the precedent to introduce, rapidly, a combination partner to work alongside DFMO has been set.

Our investigation into combination therapies against *T. brucei* focused on a group of enzymes (denoted as Group A) that, when used in tandem with weak perturbation of other enzymes (denoted as Group B), result in a similar or even more potent inhibitory effect than when these other enzymes alone (Group B) are strongly perturbed. Knowledge gained from this kind of combination therapeutic schemes on how potent a compound needs to be perturbed in order for it to be an effective drug target helps look for alternative solutions when some enzymes cannot be strongly inhibited. We found through model simulations that MAT and OrnPt are good candidates to be taken as Group A enzymes. The following section provides a detailed analysis of the results.

**Figure 9:**
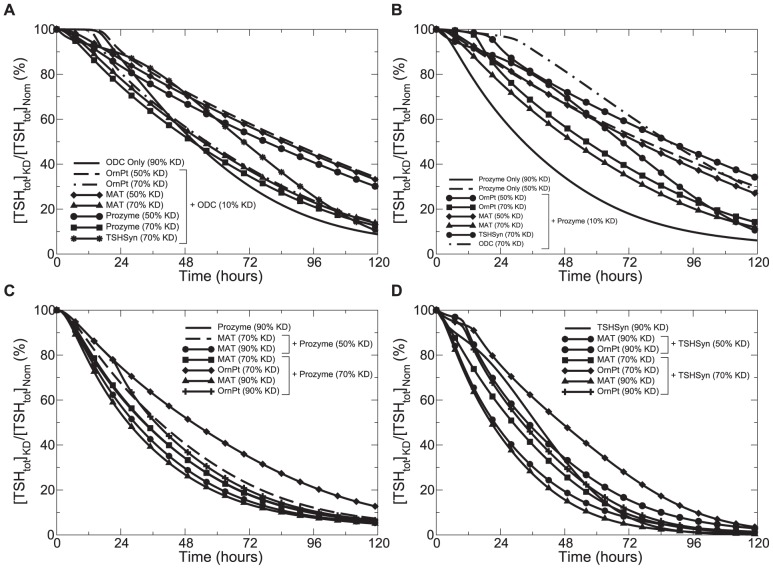
Studies of combination chemotherapeutic regimens. Percentage of 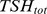 concentration under perturbed (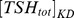, over a simulated time span of 5 days) and normal (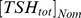) conditions. In individual model simulations (A) and (B), a 10% enzyme knockdown (KD) of ODC and prozyme is applied in conjunction with down-regulation of other key pathway enzymes and the simulation results from individual and combined perturbations are compared. In (C) and (D), the inhibitory effects on 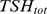 were examined for combinations of medium to strong depression of prozyme and TSHSyn, respectively, with different levels of knockdowns of other enzymes. In all cases, the maximum velocity of each enzyme is a time-dependent variable subject to specific inhibition within 24 hours.

Studying effects of inhibiting pairs of enzymes on 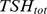, as illustrated in Figure 9A, shows that a combination of a 70% knockdown of enzyme MAT, prozyme or OrnPt with a weak (10%) down-regulation of ODC produces a similar effect on 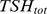 depletion as when ODC is almost completed removed. In conjunction with a 50% loss of MAT, prozyme or OrnPt, a weaker 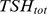 inhibition is obtained at the end of a simulated time span, but a faster depletion rate is displayed over the first 24 hours of inhibition than using a 90% ODC knockdown alone. A 10% prozyme depression (Figure 9B), together with a 50% down-regulation of MAT or OrnPt decreased 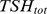 concentration to the same level at the end of a simulated time span as when only a 50% prozyme depression was applied. In conjunction with a 70% MAT or OrnPt down-regulation, the same 10% prozyme knockdown decreased 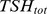 to the same extent as a 90% prozyme knockdown alone. Furthermore, combining the same 10% prozyme knockdown with a 70% loss of ODC resulted in the same degree of 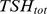 depletion as lower-level joint perturbations (50%) with MAT or OrnPt. In individual cases, combining a 10% knockdown of ODC or prozyme with a 70% TSHSyn inhibition depleted 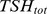 to the same amount as when the respective enzyme is perturbed by 90%, but with a slower inhibitory trajectory compared to combination therapies with MAT and OrnPt.

Figure 9C and 9D support our previous conclusion that TSHSyn and prozyme alone are capable of adequately removing 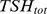 when they are subject to a sufficiently strong deactivation. As indicated in Figure 9C, the combination of a down-regulation of 70% in prozyme with a 70% depression of enzyme MAT produces the same temporal dynamics and final depletion of 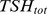 as a 90% prozyme knockdown alone. The maximum level of 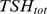 depletion occurs when prozyme (knocked down by 50% or 70%) is combined with a more potent 90% MAT down-regulation. Combining a 70% loss of prozyme with a medium to strong OrnPt perturbation can lead to a similar level of 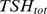 depletion, but not as strong as exerted by MAT. In Figure 9D, when TSHSyn is down-regulated by more than 50%, down-regulation of MAT or OrnPt by as much as 70% is required in tandem to obtain the same level of 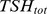 depletion as a 90% TSHSyn down-regulation alone. We observed that even though combination therapies for TSHSyn and prozyme result in approximately the same level of 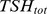 depletion at the end of the simulated time span, they exhibited faster inhibitory trajectories, giving rise to more 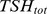 removal at earlier stages (the first 2–3 days of simulation span, see Figure 9D). In both cases, combining a 70% knockdown of prozyme or TSHSyn with a 70% OrnPt down-regulation led to the same final 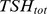 level, but with slower temporal dynamics than other strategies over the same duration.

As indicated in Figure 8, MAT and MetPt knockdown both result in almost the same depletion pattern for 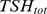. As such, MetPt related perturbation was found to be applicable to the perturbation experiments carried out here in the same way as MAT. It has been verified that a constant supply of Met is imperative for trypanosomal cell growth [24], [25], supporting the credibility of the predictions made by this model. Similarly, the results observed for ODC are applicable to SpdS, however SpdS displayed a better inhibitory effect than ODC (likely due to the regulatory link predicted for the enzymes) but still not comparable with that from MAT or OrnPt.

The combination chemotherapeutic strategy suggests that enzymatic reactions of AdoMet production and Orn uptake, catalysed by MAT and OrnPt, respectively, are key regulatory points in the pathway. When used alone or in tandem with weak down-regulation (i.e. 10%) of other enzymes, a moderate perturbation (i.e. 50%) of MAT and OrnPt exhibited a strong inhibitory impact on the total trypanothione production, with the former being more effective than the latter, in particular, when MAT knockdown is used in conjunction with medium or strong perturbation of prozyme and TSHSyn. The regulation of polyamine synthesis via MAT or OrnPt is likely to be a good chemotherapeutic target.

### Relation to *T. cruzi* model

Our polyamine model complements a recent attempt at modelling trypanothione (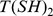) metabolism in the related parasite *T. cruzi* [6]. The *T. cruzi* model focuses on the glutathione synthesis branch and the redox cycle of 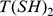. Polyamine synthesis, which is the focus of this work, is not included. Our simulation results (Figure 8) agreed with observations made in the *T. cruzi* model that at 80%–100% down-regulation, most of the involved enzymes are found to be essential for parasite survival. In particular, TSHSyn (TryS in the *T. cruzi* model) has to be inhibited by 70% to sufficiently deplete total trypanothione contents which is consistent between the two models.

Both studies attempt to identify promising therapeutic strategies and this issue is viewed from the aspect that “suitable drug targets should be enzymes for which low pharmacological inhibition have a high impact on pathway function [6]”. Pathway enzymes in the *T. cruzi* model were ranked according to control efficiency of individual enzyme and simultaneous inhibition of those enzymes with top scores were recommended as being good candidates for multi-target strategies, whereas in our *T. brucei* model, different combination therapies of key pathway enzymes were simulated and time-dependent concentration changes were measured against total trypanothione contents (Figure 9), providing us with a direct comparison among alternatives. We would like to take this work further by merging these two models to evaluate the perturbation effect on total trypanothione contents when the good targets identified from the respective work are jointly used. However, this is challenged considerably not only by the differences in parameter values but also the kinetic reactions specific to individual organisms. For example, the cysteine uptake reaction that was not modelled in the *T. cruzi* model has proven to be critical for trypanosomal survival in *T. brucei* [25]. Compared with *T. brucei*, *T. cruzi* lacks ODC activity and relies on Put uptake from the extracellular medium. Additionally, both organisms can synthesise Spd *de novo* from dAdoMet and Put, but *T. cruzi* also has the capability to assimilate exogenous Spd (this uptake reaction was modelled as the only source of endogenous Spd in the *T. cruzi* model). Integration of these models could further assist in gaining an in-depth understanding of the overall metabolic system in trypanosomes.

## Conclusion

Here we present the first model of a second branch of metabolism, the polyamine pathway, which can be linked to an existing model of glycolysis via the second route of glucose metabolism in *T. brucei*, the pentose phosphate pathway that creates NADPH, which is the ultimate source of electrons required to form the reduced trypanothione (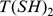) and the cell's primary reactive thiol species.

This mathematical model provides continuous and deterministic descriptions of system dynamics by applying ODEs, which has previously been employed to model quantitatively the glycolysis pathway in bloodstream-form *T. brucei* [26]. An alternative approach to modelling metabolic systems is via structural modelling. Structural modelling takes the stoichiometry and reversibility of chemical reactions as the only inputs, which is in contrast to kinetic modelling where precise information of involved enzymatic rate equations and associated parameter values is a prerequisite. Structural modelling is a relatively straightforward process and because the knowledge required for this approach is primarily the stoichiometry of a system, the drawback is the limited predictive power in studying system dynamics that involves manipulating enzymatic mechanisms. Therefore, structural modelling is often regarded as a precondition for kinetic modelling.

Our modelling activities focused on studying the effectiveness of DFMO, the first line drug licensed to target stage 2 HAT. Previous work has generated a significant amount of information regarding the network topology and kinetic analysis of many of the enzymatic reactions has made kinetic modelling possible. However, parameters for a significant number of the enzymes involved in the pathway were unknown. Therefore it was necessary to introduce assumptions and simplifications to the pathway were required. Qualitative knowledge of the pathway guided the assumptions made and optimisation-enabled dynamic simulations were used to test how assumption-containing models performed relative to outputs measured in experiments. Discrepancies between model simulations and experimental observations prompted a cyclic procedure of model design. The mathematical formulation of the model equations together with the estimated set of parameters faithfully reproduces most experimentally measured properties of the pathway.

The model already offers opportunities to explore new strategies for targeting this pathway in anti-trypanosomal drug design. Combined down-regulation of key pathway enzymes offers an effective chemotherapeutic strategy. Combination chemotherapeutic studies revealed that most polyamine enzymes can influence polyamine biosynthesis, but when targeted alone, high levels of inhibition are required to inhibit the pathway sufficiently to kill cells. Most importantly, reactions catalysed by enzyme MAT or OrnPt appear to be critical control points of the pathway, with MAT being preferable to OrnPt. Moderate disruption of MAT or OrnPt, both in isolated and joint form, led to dramatic changes in polyamine concentrations and total trypanothione contents. Our study also shows that prozyme and TSHSyn could be used for multi-target therapy but only when they are potently inhibited (at least 50% knockdown) together with similar down-regulation of MAT or OrnPt.

In general, enzymes or metabolites identified in parasites and known to be absent from or significantly different in the mammalian host were ideal targets for chemotherapy. In *T. brucei*, MAT is insensitive to control by product inhibition of AdoMet but mammalian isoforms of this enzyme are highly sensitive to AdoMet. The function of MAT in linking inhibition of polyamine synthesis to disruption of AdoMet metabolism and the differences of MAT in host and parasites could make this enzyme a critical drug target. *T. brucei* lacks arginase and depends on efficient Orn uptake, which makes OrnPt an especially attractive drug target. Certainly, a valid target should not only be lethal to parasites but also be acceptably safe for human patients in long-term clinical usage. Therefore, these potentially good drug targets have to be further validated in terms of the therapeutic benefit and safety.

In conclusion, it has been necessary to include multiple assumptions and simplifications to build a model of polyamine metabolism in *T. brucei* because insufficient data was available to produce a full description. Notwithstanding, the availability of several datasets giving measurements of metabolite levels following pathway perturbation has enabled us to adjust assumed parameters and simplifications in a way that allows reasonable simulations of measured activity. The model has then been used to make predictions on potential co-inhibition of different enzymes of the pathway to inform possible strategies for combination chemotherapy and can report on possible regulatory components of the pathway which can now be approached experimentally. The basic model description here can be further improved as new information becomes available in *T. brucei* on specific kinetic parameters of enzymes in the pathway and measured metabolite levels under different perturbed conditions.

## Materials and Methods

### Considerations for model construction

van Riel [27] argued that many attempts of computational modelling pursue realistic large-scale complex models, but very often simplified models are feasible and at least as valuable in understanding the essential features of biological systems. The following considerations were made in order to study the polyamine pathway in isolation from the entire network as presented in Figure 1.

Firstly, the involvement of the trans-methylation branch (responsible for the production of cystathionine via homocysteine) was limited to the first step describing the conversion of AdoMet into AdoHcy (S-adenosylhomo-cysteine). As observed in [28], [29], metabolic products of trans-methylation reactions (i.e homocysteine and cystathionine) are mostly secreted from trypanosomal cells, which leave their contributions in polyamine biosynthesis and regulation very minimal. Parasitic *T. cruzi* and Leishmania species lack the enzyme of Met synthase, which catalyses the Met production from homocysteine; however, debate remains as to whether homocysteine can be converted to Met in *T. brucei* [30]. Goldberg et al. [31] also suggested that, even though homocysteine remethylation may exist in *T. brucei*, as most trans-sulfuration metabolites are secreted from trypanosomes, any homocysteine recycled to Met will not be significant. AdoHcy, which is toxic if accumulated in cells [28], was also observed to remain unchanged under perturbed conditions in *T. brucei*, i.e. during 36 hours of DFMO treatment [29], and thus is treated as a constant metabolite in our study.

Secondly, we excluded glutathione biosynthesis and related reactions from consideration and modelled the biosynthesis of 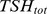 with a single-step reaction from Spd, catalysed by a synthetic enzyme, named TSHSyn. In *T. brucei*, the reduced trypanothione, 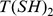, is synthesised in two steps. First, a single molecule of Spd is combined with glutathione to generate a glutathione-spermidine conjugate (not shown in Figure 1). This is followed by the addition of a second glutathione creating the reduced trypanothione from. It has been reported that both synthetase and amidase activity are associated with biosynthesis of the reduced trypanothione in *T. brucei* [32] as well as in *Leishmania* parasites [33] and *Crithidia fasciculata* [34]. The conflicting activities of synthetase and amidase allow for a bidirectional response between the involved metabolites, which may serve to modulate intracellular levels of these metabolites without additional biological processes (i.e protein synthesis or degradation of existing metabolites). There is however very limited information for enzyme kinetics of the intermediate steps of glutathione biosynthesis and the regulation mechanism between synthetase and amidase has not yet been precisely characterised in *T. brucei*. The approximate description of 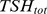 biosynthesis reduces the degrees of freedom and diminishes the impact of unknowns in the model simulations. In the rate equation of TSHSyn, a regulatory term for the reaction product 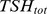 is included to reflect the self-regulation ability of Spd and 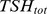, as if they are modulated by the amidase activity.

Finally, the link between 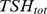 and the remaining system (i.e. the pentose phosphate pathway in the grey box in Figure 1) was modelled as a black box (refer to [35] for a case study). Black-box modelling is a popular approach for modelling chemical processes that lack physical insight or are highly abstract [36], [37]. The key of this approach is to approximate input-output dynamics of the involved metabolites. In our work, this is the relation between Spd (input) and trypanosomal growth (output, proportional to the concentration of total trypanothione, 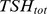). This modelling strategy facilitates the practical construction of a useful model of polyamine metabolism with predictive capabilities; this can only be achieved when all intra-cellular metabolites are modelled as time-dependent variables. Black-box structures are parameterised descriptions, which can be approximated in the form of, for example, power series polynomials and fuzzy logic. In this study, a combination of a Hill equation and a linear decay function proportional to the concentration of 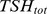 is used to model this reaction (details are given in the Model Descriptions section of Materials and Methods). Inclusion of total trypanothione in the model allows us to quantify explicitly the consequences of polyamine interruption on cell growth arrest in the context of the model.

### A summary of iterative model design

In the standard approach (see [38], [39] for details), an initial model topology that approximates the input-output relationship of the system is constructed, and then a parameter estimation process is applied to match a particular dataset against model structure. Once a candidate model is built in this way, it can be tested on validation data, i.e. data not used in the parameter estimation step. If the estimate-containing model demonstrates predictive power it may be considered to be relevant in describing the underlying processes. Where inconsistency emerges between model predictions and experimental observations the model is refined and iteratively evaluated against validation data.

Following the above system identification procedure, five candidate models were generated, which share the same topology but differ with respect to the mathematical representations of enzyme kinetics. Refinements made throughout model construction were summarised in Table S2, where the final model performed the best on both the estimation data and validation data and its description form the basis of this paper.

### Model descriptions

The polyamine biosynthetic pathway is described mathematically by eight ODEs (Table 2), each of which corresponds to a time-dependent variable metabolite. The ODE model takes exogenous methionine (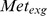) and ornithine (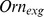) as the only inputs, since *T. brucei* does not have an efficient mechanism for the assimilation of exogenous putrescine (Put) and Spd, and relies on *de novo* synthesis to acquire these two polyamines [15], [40]. Concentrations of both external (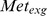 and 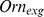 in blood) and constant (AdoHcy) metabolites are fixed at their physiological levels.

**Table 2:** Differential equations for the time-dependent variables included in the model.

Variables	Differential Equations
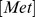	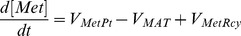
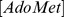	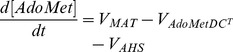
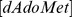	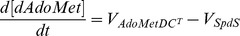
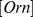	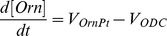
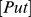	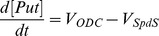
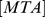	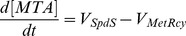
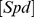	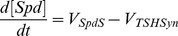
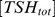	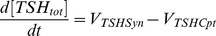

Rate equations for individual enzyme-catalysed reactions, which are displayed on the right hand side of the ODEs, are detailed below.

**ODC** catalyses the initial step in the pathway leading to Put production from Orn. ODC has an extremely short intra-cellular half-life in mammals, reportedly 15 min to 1 hr, which is in contrast to the more stable protein in *T. brucei*, which has a turnover rate greater than 6 hrs. The reversible rate law was applied to model ODC kinetics in the form below, which is subject to weak product inhibition by Put and postulated correlation of SpdS on ODC. Due to the lack of information on these two parameters, we assumed the half-saturation constant 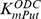 to have the same value as the known parameter 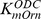 and we analytically derived the equilibrium constant 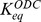 from the experimental observations of AdoMetDC RNAi induction and prozyme knockout. Refer to Table S2 and Text S4 for more information on these two parameters. When SpdS remains uninduced, parameter 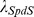 is zero and thereby the maximum velocity of ODC becomes time-independent. Under SpdS perturbations, a positive value has been deduced for parameter 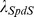 from the given inhibitory profile of SpdS deactivation to mimic the temporal changes of SpdS activity over time and in this case the maximum velocity of ODC becomes time-variant and influenced by the activity changes of SpdS.
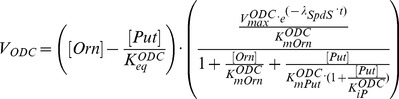
(1)
**AdoMetDC** is responsible for the formation of dAdoMet, the aminopropyl donor for the biosynthesis of Spd from Put. As is the case for *T. brucei* ODC, *T. brucei* AdoMetDC is a stable enzyme and has a lower turnover rate than in mammalian cells. AdoMetDC is also a regulatory enzyme, regulated by an allosteric mechanism with prozyme, which is an enzymatically inactive close homologue of AdoMetDC itself. The regulation of AdoMetDC is induced by a conformational change of the prozyme structure, which alters the half-saturation constant of AdoMetDC activity. Willert et al. [41] discovered that in *T. brucei* neither AdoMetDC nor prozyme *per se* is sufficiently active to prompt normal cell growth, and only the complex of AdoMetDC—prozyme can maintain the physiological level of Spd. Recent work by Willert and Phillips [16] has extended the subject to examining the influence of AdoMetDC RNAi inhibition and prozyme knockout on polyamine synthesis and parasite growth. A similar mechanism of allosteric regulation was also found for *T. cruzi* AdoMetDC [42].

The binding of AdoMetDC with prozyme contributes to dynamical control of metabolic fluxes in the polyamine pathway [41]. We represent the enzyme-ligand binding between AdoMetDC and prozyme as a one-step conformation system, with the plausible assumption that the ligand can interact rapidly with the enzyme as prozyme concentration is not comparable with AdoMetDC concentration [41], causing the reaction to occur at a rapid equilibrating rate following linear mass action kinetics (i.e. 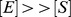). Because prozyme levels are restricted, AdoMetDC is present in trypanosomal cells in both ligand-occupied form and free form. Accordingly, we express the velocity equation of the total AdoMetDC as a superposition of two terms stemming from the individual forms of the enzyme, as below. The representation of regulatory capabilities in summation of distinct states has been verified for allosteric enzymes in [43].
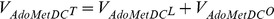
(2)where
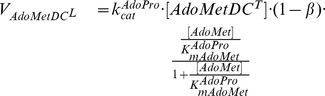


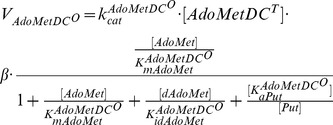
In these equations, 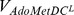 and 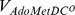 stand for the velocity contributed by the ligand-occupied (binding with prozyme) and free form of the enzyme, modelled as above. A factor 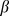 represents the percent of free-form AdoMetDC (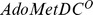) taking up the total enzyme concentration (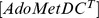), thus the ligand-occupied form (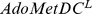) is expressed as 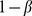 of the total concentration. Since the prozyme concentration is smaller than that of AdoMetDC [16], 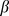 is assumed to vary between 0.5 and 1 in order to reflect the experimental observation and still allow the ligand-occupied AdoMetDC to change within a physiologically feasible range. Note that in the above rate equations, Put and dAdoMet have a stimulatory and inhibitory effect respectively on the activity of free-form AdoMetDC but not on the AdoMetDC—prozyme heterodimer (the ligand-occupied form) [41]. *T. brucei* AdoMetDC was thought to be insensitive to dAdoMet, which is in contrast to the strong product inhibition exerted by its counterpart in many other species (e.g. mammalian cells) [19]. A wide range of 1 to 1000 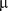
M is applied for the parameter 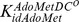 and the estimate from *in silico* simulations can be used to qualitatively assess the contradictory report for this parameter.

**MAT** catalyses production of AdoMet from Met in the presence of ATP. AdoMet plays an important role in a variety of cellular functions, such as methylation and sulphuration. Polyamines are not inhibitory to the enzyme within the range of 10 to 5000 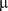
M, and positive cooperativity was only realised at higher concentrations of ATP with a Hill constant (*nMAT*) equal to 2.0 [44]. In our model, ATP was regarded as a constant metabolite due to the lack of knowledge on its uptake kinetics; this is supported by the recent work [6] that the concentration of ATP stays high and constant under stress and non-stress conditions. The enzyme velocity is modelled in the form below, where AdoMet exerts only a weak inhibition on MAT, which is competitive with respect to the substrate Met.
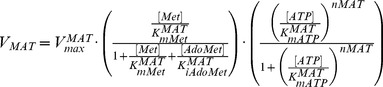
(3)
**SpdS** catalyzes Spd biosynthesis from Put in the presence of dAdoMet, with methylthioadenosine (MTA) as a by-product. MTA is not detectable in mammals because of its rapid degradation rate [3], [45], which gives rise to the intra-cellular concentration of this compound being low [46]. Since no data is available for the physiological level of MTA in *T. brucei*, according to the observation in mammalian cells, MTA is assumed to hold a small value of 20 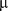
M in our study. The kinetic mechanism of this enzyme is modelled below, subject to the product inhibition [3], [47]. Note that when SpdS remains wild-type, parameter 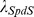 is 0, and thereby the maximum velocity of SpdS becomes time-independent. Under perturbed conditions, the maximum velocity of SpdS becomes time-variant and defined in accordance with the value of parameter 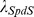.
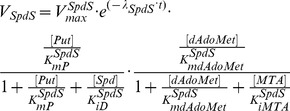
(4)
**MetRcy** catalyses the synthetic transition from MTA to Met. MTA is recycled to Met via a series of enzymatic steps in trypanosomes [48]. It is first converted to methylthioribose-1-phosphate by MTA phosphorylase; the latter product is then metabolised to keto-methylthiobutyrate, and ultimately to Met [49]. Because of the importance of MTA recycling in cell viability, interference with Met metabolism has been explored as a potential drug target in mammals and *Plasmodium falciparum* [24], [50], [51]. In mammalian cells, Met can be regenerated via enzymatic catalysis of homocysteine [50]; however debate remains as to whether homocysteine remethylation exists in *T. brucei*, given that the enzyme catalysing this chemical transition is absent in other related parasitic species (e.g. *T. cruzi* and *Leishmania*) [30].

In our study, the MTA recycling path is considered as the unique source of Met reproduction, which is assumed to occur via a single-step reaction, as kinetics for the intermediate reactions are not known experimentally. In *T. brucei*, available quantitative descriptions for the recycling path are limited to the half-saturation constant of MTA phosphorylase with respect to its substrate MTA. Since the enzyme has a broad substrate specificity [52], the *in vivo* maximum velocity is hard to obtain, but it is assumed to hold a very high value [53]. Again, standard Michaelis-Menten kinetics are applied to describe the enzyme kinetics, shown below:
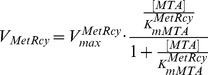
(5)
**AHS** catalyses the production of AdoHcy from AdoMet. The enzyme velocity is modelled as follows, subject to strong product inhibition by AdoHcy [54].
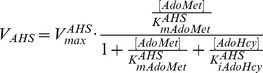
(6)AdoHcy is regarded as a constant metabolite during the *in silico* simulation and a methylation index of 2∶1 [28] is assumed for the ratio of [AdoMet] to [AdoHcy] under wild-type conditions (resulting in the constraint [AdoHcy] = 0.5

[AdoMet]) to approximate the relationship between the concentrations of the metabolites.

**TSHSyn** denotes the synthetic enzyme catalysing one-step 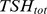 production from Spd in the model. We employed an irreversible Hill equation (with *nSyn* standing for the Hill coefficient) to model this enzyme, which is characterised by competitive product inhibition by 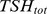, shown as follows. This kinetic structure allows the model to mimic the *in vivo* state where 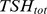 levels can be compensated by elevating its production rate during *T. brucei* growth interruption (via reducing 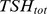 level).
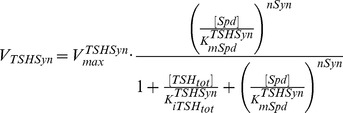
(7)
**TSHCpt** denotes the sink reaction responsible for 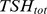 interactions with the remaining system, in order to prevent unrestricted accumulation. Designing a suitable expression for this abstract enzyme is challenging. A linear function of consumption rate (an unknown parameter) multiplying the concentration of total trypanothione was initially proposed as the minimum consumption requirement; however the simulated behaviour of 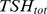 failed to reproduce either the steady-state or DFMO-perturbed data. We refined the rate definition by adding to this function an irreversible Hill equation representing enzyme-catalysed breakdown of the metabolite. Due to the number of existing unknowns, we approximated the consumption rate (in the linear function) as the specific growth rate (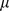) to which the consumption of total trypanothione is proportional in reality. This combined expression later proved to be satisfactory for total trypanothione, and simulations converged to the expected steady state, with rapid consumption under perturbation conditions, as shown below.
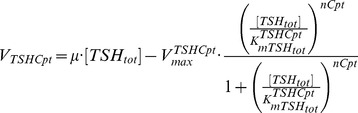
(8)The current representations for rate equations of TSHSyn and TSHCpt are capable of reproducing observed behaviour of total trypanothione (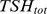) under various experimental conditions. These kinetic structures will inevitably be different when the remaining metabolites in the network are integrated into this model.

**MetPt** is responsible for the uptake of exogenous Met in our model. Trypanosomes rely on a constant supply of Met, and *de novo* synthesis is energetically expensive [24], [25]. Standard Michaelis-Menten kinetics are applied to model MetPt as below
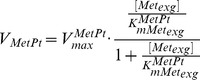
(9)
**OrnPt** is responsible for the uptake of exogenous Orn, which is modelled based on the reversible Michaelis-Menten kinetics. Exogenous Orn is considered as a constant supply into the system, with the plasma concentration assumed to be 77 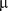
M [13]. Parameters 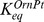
and 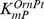 stand for the equilibrium constant and the half-saturation constant of product Orn, respectively.
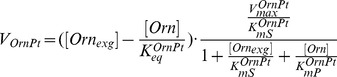
(10)This ODE model of polyamine metabolism contains 40 kinetic parameters, where 20 are unknown and two are solved analytically. To ensure unit consistency of the model parameters, we express all wild-type maximum velocities 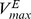 (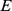 refers to specific enzyme name) in units of 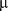
M per minute and hence in all these rate equations, the derivatives of the concentrations (d[Metabolite]/dt) are expressed in 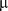
M per minute. Some known enzyme velocities were measured in different units, i.e. 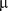
mol per minute per number of cells or per mg of protein, conversion of which into the desired unit was required before carrying out model simulations. Unit conversions are elucidated in Text S2.

### Model calibration

Model calibration involves determination of model parameters that can reproduce the system behaviour. A common procedure is to first fit model parameters to experimental data generated by a reference cell type (wild type) and then test the estimates on data generated by a variation (mutant). In our study, we adopt a novel estimation methodology - the multi-objective optimisation algorithm MoPSwarm [55] - to estimate unknowns, where both the steady-state (wild type) and the perturbed (drug treated or genetic mutant) conditions of the pathway are handled simultaneously. It has been demonstrated in [55] that accounting for more than one state of the system in parameter estimation process is an advantageous approach for obtaining reliable parameter estimates.

In this study, the model was trained via simultaneous fitting against both the physiological steady state and DFMO-mediated inhibition. However, corrections had to be made before the dataset can be used. For example, AdoMet levels in trypanosomes during ODC inhibition by DFMO treatment, were reported as being elevated 75 times by Fairlamb et al. [11] whilst levels of this metabolite were almost unchanged during DFMO treatment studied by Xiao et al. [14]. The parameter estimation process was applied to the model to match the estimated data with an increased AdoMet concentration. Simulation results, however, predicted that AdoMet contents were largely unchanged and all other metabolites were well fitted. Thus, for model calibration, we replaced AdoMet behaviour (considerably increased) in Fairlamb's data set with constant dynamics (as observed by Xiao et al.); dynamics of all other metabolites in Fairlamb's data set remained intact.

We used the temporal changes of the reduced trypanothione, 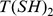, to approximate the dynamics of total trypanothione concentration in our model. This is because in the work by Fairlamb et al., and in all other perturbation experiments, measurements were made for the reduced trypanothione only. Since the reduced trypanothione exists in much higher concentrations than the oxidised trypanothione in *T. brucei*, the reduced trypanothione is taken to represent the trends in total trypanothione changes over time.

The polyamine model under steady-state (wild type) and DFMO-treated (perturbed) conditions differ in the mathematical representation of 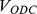, as the maximum velocity of ODC (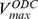) is a time-invariant parameter in the former case and a time-dependent exponential decay in the latter. Uptake kinetics of DFMO have not been measured. Despite the absence of a quantitative description, the DFMO-induced inhibition is well understood in a qualitative sense, where ODC activity decreased by more than 99% within 12-hour of treatment with DFMO [11]. ODC activity in response to DFMO is therefore modelled with an exponential decay function by multiplying the original rate equation of ODC (Equation 1 in the Model Descriptions section of Materials and Methods) with term 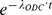 to reflect the time-dependent response of enzyme activity to the drug inhibition, whilst remaining terms in the equation are unchanged, shown below. Parameter 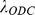 takes a value of 0.007 in this instance, solved by simple curve fitting using the qualitative description.
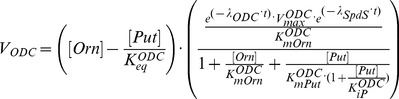
(11)Note that in this equation parameters 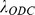 and 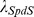 correspond to different inhibitory scenarios, namely ODC inhibition (resulting from DFMO drug uptake or ODC enzyme perturbation) and SpdS inhibition, respectively. It is only when ODC and SpdS inhibition are applied in tandem that both parameters are given non-zero values. In other words, except in SpdS-inhibited conditions, 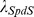 equals zero under both steady-state and DFMO-induced conditions as well as all other perturbed conditions.

In our model, initial concentrations are treated as unknown parameters to be estimated together with the unknown kinetic parameters. Our choices of the initial metabolite concentrations are restricted to 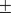
20% of the measured physiological levels when fitting the polyamine model to the given steady state. This helps the convergence of the optimisation algorithm from random positions in the search space. The solutions returned from the estimation procedure are ranked according to their importance in satisfying both pathway states using the root mean square of the two objectives with respect to an individual state, and the best trade-off solution with the highest rank is selected for investigation. Details on the optimisation algorithm, the objective functions and the ranking method used for parameter estimation and selection are given in Text S3.

## Supporting Information

Table S1 **Estimates of the initial concentration values of polyamine metabolites.**
(PDF)

Table S2 **Summary of model refinement process.** Descriptions and diagnosis of the different versions of the model.
(PDF)

Figure S1
**Acquisition of steady state under perturbation of the initial polyamine concentrations.** The kinetic model was simulated with the initial condition of polyamine concentrations varied by up to 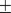
80% of the estimated values (Table S1) while maintaining all the kinetic parameters unchanged. Simulation results showed that the model can converge to exactly the same *basal* condition with different initial values over a simulated time span of 4 days, indicating no numerical artefacts contained in the model.(EPS)

Figure S2
**Time-series simulation of TSHCpt inhibition on** 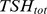 **level.** During the simulation, the maximum velocity of TSHCpt was modelled as a time-dependent variable using the exponential decay function with 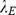 set to 0.00045, which inhibited the enzyme activity by 90% within 3 days.(EPS)

Text S1 **Kinetic parameters of the model.** Reports of extracted literature values and estimates of empirically-derived boundaries for the unknown parameter space.
(PDF)

Text S2 **Unit consistency for kinetic parameters.** Explanations on converting different units of maximum velocities into a universal unit.
(PDF)

Text S3 **Parameter Estimation and Selection.** Design of objective functions and selection of the best set of estimates.
(PDF)

Text S4 **Analysis on the Model Refinements.** Impact of the reversibility of enzyme ODC and the regulatory effect of SpdS on model behaviours.
(PDF)
