# Supplementary material for: Mathematical Modelling of Polyamine Metabolism in Bloodstream-Form Trypanosoma brucei: An Application to Drug Target Identification
Source: PLoS One. 2013 Jan 23;8(1):e53734. doi: 10.1371/journal.pone.0053734 (PMC3553166; doi:10.1371/journal.pone.0053734)
Supplement: Table S1 — Estimates of the initial concentration values of polyamine metabolites. (PDF) [file pone.0053734.s001.pdf]

Table S1. Estimates of the initial values of polyamine concentrations.

**Metabolites**	**Met**	**AdoMet**	**dAdoMet**	**Orn**	**Put**	**MTA**	**Spd**	TSH_{tot}
**Values** (μ**M**)	3038.4	17.7	7.7	51.6	620.4	17.4	1655.2	325.1
