# Supplementary material for: Mathematical Modelling of Polyamine Metabolism in Bloodstream-Form Trypanosoma brucei: An Application to Drug Target Identification
Source: PLoS One. 2013 Jan 23;8(1):e53734. doi: 10.1371/journal.pone.0053734 (PMC3553166; doi:10.1371/journal.pone.0053734)
Supplement: Table S2 — Summary of model refinement process. Descriptions and diagnosis of the different versions of the model. (PDF) [file pone.0053734.s002.pdf]

Table S2. Summary of model refinement process.

Version	Descriptions	Results
V1	We modelled all enzymes on the basis of standard irreversible Michaelis-Menten (MM) kinetics and incorporated the regulatory effect of production inhibition where specified in the literature.	Model failed to fit Orn, Spd and TSH_{tot} dynamics (i.e. the trend of concentration changes). For example, within 12 hours of DFMO treatment, Orn linearly increased to almost 30 fold of the initial value, which was reported as having an exponential approach in the estimation data. Spd and TSH_{tot} were reduced by a small amount only, which disagrees with the considerable depletions reported in the literature.
V2	We modelled the enzyme catalyzing Orn uptake (V_{OrnPt}) with reversible MM kinetics, as we suspected that rate descriptions of Orn-centered reactions may be ill-characterized, but there is no available biological knowledge about *T. brucei* that can be used for postulating the rate equation. We introduced two additional parameters K_{eq}^{OrnPt} (equilibrium constant) and K_{mP}^{OrnPt} (Michaelis constant of product) associated with the reverse component of the reaction and estimated them along with existing unknown parameters.	Orn dynamics under DFMO treatment were significantly improved in terms of the trend in transient concentration changes in comparisons to Model V1. No obvious improvements were seen for Spd and TSH_{tot} from the changes imposed on the rate expression of Orn uptake. The enzyme responsible for the reaction between Spd and TSH_{tot} may be ill characterized.
V3	Since TSH_{tot} production from Spd is highly abstract in our system, selecting an appropriate function to approximate kinetics for TSHSyn is not straightforward. We refined enzyme kinetics catalyzing TSH_{tot} synthesis by adding a regulatory term representing product inhibition by TSH_{tot} to the irreversible MM kinetics used previously. This ensures resources not devoted to making TSH_{tot} when it is plentiful.	Simulation results agreed with the estimation data in terms of the trend in transient concentration changes for all metabolites under DFMO treatment. Together with the best set of parameter estimates, the model can also reproduce most of the validation data except Put dynamics when AdoMetDC, prozyme and SpdS are inhibited. A considerable build up in Put concentration was predicted in all cases, which is counter to experimental observations that Put does not significantly accumulate over time.
V4	On the basis of Model V3, we postulated an active regulation of SpdS on ODC through trial-and-error simulation experiments. This regulation in the expression of $e^{(-\lambda_{SpdS} \cdot t)}$ prevents Put accumulation by restricting Put production rate when its consumption rate is largely reduced.	Put accumulation in response to SpdS perturbation is avoided, but in the cases of AdoMetDC RNAi induction and prozyme knockout, Put level still continuously increased over time unexpectedly.
V5 (final model)	On the basis of Model V4, we refined the ODC-catalyzed reaction with the reversible rate law according to the postulation made in [17] that Put may be simply converted to Orn. We employed the parameter estimates from model V4 and analytically derived values for the newly-added parameters K_{eq}^{ODC} and K_{mPut}^{ODC}.	Put level increased within 24 hours of the inhibitory induction of AdoMetDC or prozyme and then gradually reached a plateau. This model properly fits estimation data and reproduces all the validation data well, providing evidence of the validity of the model.
