# Supplementary material for: Mathematical Modelling of Polyamine Metabolism in Bloodstream-Form Trypanosoma brucei: An Application to Drug Target Identification
Source: PLoS One. 2013 Jan 23;8(1):e53734. doi: 10.1371/journal.pone.0053734 (PMC3553166; doi:10.1371/journal.pone.0053734)
Supplement: Text S1 — Kinetic parameters of the model. Reports of extracted literature values and estimates of empirically-derived boundaries for the unknown parameter space. (PDF) [file pone.0053734.s005.pdf]

Supplementary text 1: Kinetic parameters of the model

Table below contains enzyme parameters extracted from the literature, where unknown parameters were either estimated against DFMO-induced polyamine profile (the best trade-off solution is selected) or measured experimentally. Ranges of the unknowns are empirically derived with measured kinetic parameters as references, since no guidance or rules exist that can help with this process. In our study, a range of 0 and 1000 μM is assumed for most half-saturation constants K_m, which covers all the K_m where experimental data is available (except for K_{mATP}^{MAT} which is over 1000 μM and it may be due to the high concentration value of ATP (4000 μM) this parameter is with respect to; in our model no other metabolite exists in such high concentration). For K_{mSpd}^{TSHSyn}, $K_{iTSH_{tot}}^{TSHSyn}$ and $K_{mTSH_{tot}}^{TSHCpt}$ which are involved in the rate equations of the abstract enzyme TSHSyn and TSHCpt, a relatively wider range is assumed (0 to 3000 μM) to compensate for the exclusion of related metabolites and to assist the optimizer in exploring possible solutions. The same consideration is applied for deriving the ranges for the hill constants and equilibrium constant. As a range of 1e-4 to 1e-2 was observed for enzyme ODC, SpdS, MetPt and OrnPt, a range of 1e-6 to 1 μmol per minute per mg of protein is assigned to unknown V_{max}^{E} values, which extends the range of the measured maximum velocities by two orders of magnitude on each side. After applying the unit conversion (see Supplemental Text S2), this range is expressed as 0.33 to 3.34e+5 μM/min.

Table of parameter values for *T. brucei* enzymes.

Parameters	Description	Value	Source/Estimates
V_{max}^{ODC}	maximum velocity	40 μM/min	[1]
K_{mOrn}^{ODC}	half-saturation constant	280 μM	[1]
K_{iP}^{ODC}	product inhibition constant	350 μM	[2]
$k_{cat}^{AdoMetDC^O}$	turnover rate	0.078 min^{-1}	[3]
k_{cat}^{AdoPro}	turnover rate	84 min^{-1}	[3]
$K_{mAdoMet}^{AdoPro}$	half-saturation constant	110 μM	[3]
$K_{mAdoMet}^{AdoMetDC^O}$	half-saturation constant	380 μM	[3]
$K_{idAdoMet}^{AdoMetDC^O}$	product inhibition constant	1 - 1000 μM	970.64 μM
$K_{aPut}^{AdoMetDC^O}$	activation constant	1.5 μM	*Derived from* [4]
$[AdoMetDC^T]$	enzyme concentration	0.05 - 1 μM	0.63 μM
β	fractional factor	0.5 - 1	0.5
V_{max}^{MAT}	maximum velocity	0.33 - 3.34e+5 μM/min	2790.93 μM/min
K_{mMet}^{MAT}	half-saturation constant	200 μM	[5]
K_{mATP}^{MAT}	half-saturation constant	1750 μM	[5]
$K_{iAdoMet}^{MAT}$	product inhibition constant	240 μM	[5]
$nMAT$	hill constant	2	[5]
$[ATP]$	concentration in cytosol	4000 μM	*Modelling Assumption*
V_{max}^{SpdS}	maximum velocity	1057.6 μM/min	[6]
$K_{mdAdoMet}^{SpdS}$	half-saturation constant	0.09 μM	[6]
K_{mP}^{SpdS}	half-saturation constant	205 μM	[6]
K_{iD}^{SpdS}	product inhibition constant	100 μM	[6]
K_{iMTA}^{SpdS}	product inhibition constant	1e-3 - 1000 μM	0.0075 μM
$[MTA]$	intra-cellular concentration	20 μM	*Modelling Assumption*
V_{max}^{MetRcy}	maximum velocity	0.33 - 3.34e+5 μM/min	210.22 μM/min
K_{mMTA}^{MetRcy}	half-saturation constant	1 - 1000 μM	1000 μM

V_{max}^{AHS}	maximum velocity	0.33 - 3.34e+5 μM/min	1.762e+5 μM/min
$K_{mAdoMet}^{AHS}$	half-saturation constant	1 - 1000 μM	926.55 μM
$K_{iAdoHcy}^{AHS}$	product inhibition constant	12.9 μM	[7]
V_{max}^{MetPt}	maximum velocity	4965.5 μM/min	[8]
$K_{mMet_{exg}}^{MetPt}$	half-saturation constant	32.8 μM	[8]
V_{max}^{OrnPt}	maximum velocity	27.41 μM/min	[9]
K_{mS}^{OrnPt}	half-saturation constant	310 μM	[9]
K_{eq}^{OrnPt}	equilibrium constant	1 - 10	9.31
K_{mP}^{OrnPt}	half-saturation constant	1 - 1000 μM	420.86 μM
V_{max}^{TSHSyn}	maximum velocity	0.33 - 3.34e+5 μM/min	18.53 μM/min
K_{mSpd}^{TSHSyn}	half-saturation constant	1 - 3000 μM	3000 μM
$K_{iTSH_{tot}}^{TSHSyn}$	product inhibition constant	1 - 3000 μM	3000 μM
$nSyn$	hill coefficient	1 - 4	3.01
μ	specific growth rate	0.0019 min^{-1}	[10]
V_{max}^{TSHCpt}	maximum velocity	0.33 - 3.34e+5 μM/min	29.23 μM/min
$K_{mTSH_{tot}}^{TSHCpt}$	half-saturation constant	1 - 3000 μM	560.93 μM
$nCpt$	substrate inhibition constant	1 - 4	4

References

1. Phillips MA, Coffino P, Wang CC (1988) *Trypanosoma brucei* ornithine decarboxylase: enzyme purification, characterization, and expression in *Escherichia coli*. The Journal of Biological Chemistry 263: 17933–17941.

2. Osterman A, Grishin NV, Kinch LN, Phillips MA (1994) Formation of functional cross-species heterodimers of ornithine decarboxylase. Biochemistry 33: 13662–13667.

3. Willert EK, Fitzpatrick R, Phillips MA (2007) Allosteric regulation of an essential trypanosome polyamine biosynthetic enzyme by a catalytically dead homolog. PNAS 104: 8275–8280.

4. Bitonti AJ, Dumont JA, McCann PP (1986) Characterization of *Trypanosoma brucei brucei* s-adenosyl-l-methione decarboxylase and its inhibition by berenil, pentamidine and methylglyoxal bis(guanylhydrazone). The Journal of Biological Chemistry 237: 685–689.

5. Yarlett N, Garofalo J, Goldberg B, Ciminelli MA, Ruggiero V, et al. (1993) S-adenosylmethionine synthetase in bloodstream *Trypanosoma brucei*. Biochim Biophys Acta 24: 68–76.

6. Taylor MC, Kaur H, Blessington B, Kelly JM, Wilkinson SR (2008) Validation of spermdine synthase as a drug target in African trypanosomes. Biochemical Journal 409: 563–569.

7. Yarlett N, Quamina A, Bacchi CJ (1991) Protein methylase in *Trypanosoma brucei brucei*: activities and response to DL-alpha-difluoromethylornithine. Journal of General Microbiology 137: 717–724.

8. Hasne MP, Barrett MP (2000) Transport of methionine in *Trypanosoma brucei brucei*. Molecular and Biochemical Parasitology 111: 299–307.

9. Vincent IM, Creek DJ, Burgess K, Woods DJ, Burchmore RJS, et al. (2012) Untargeted metabolomics reveals a lack of synergy between nifurtimox and eflornithine against *Trypanosoma brucei*. PLoS Negl Trop Dis 6.

10. Haanstra JR, Stewart M, Luu VD, van Tuijl A, Westerhoff HV, et al. (2007) Control and regulation of gene expression. quantitative analysis of the expression of phosphoglycerate kinase in bloodstream form *Trypanosoma brucei*. The Journal of Biological Chemistry 283: 2495–2507.
