# Supplementary material for: Mathematical Modelling of Polyamine Metabolism in Bloodstream-Form Trypanosoma brucei: An Application to Drug Target Identification
Source: PLoS One. 2013 Jan 23;8(1):e53734. doi: 10.1371/journal.pone.0053734 (PMC3553166; doi:10.1371/journal.pone.0053734)
Supplement: Text S2 — Unit consistency for kinetic parameters. Explanations on converting different units of maximum velocities into a universal unit. (PDF) [file pone.0053734.s006.pdf]

Supplementary text 2: Unit consistency for kinetic parameters

Uptake rates of Met [1] and Orn [2] were given in units of nmol per minute per number of cells and by applying the relation that 10^8 cells equal to 5.8 μL in *T. brucei* [3], they are calculated as 4965.5 μM/min and 27.41 μM/min, respectively. The maximum velocity of enzyme ODC is reported for the purified native protein in the value of 45000 nmol per minute per mg of enzyme and the amount of ODC in *T. brucei* is 0.001% of the total cell protein [4]. Expressed per total cell protein, the maximum velocity of ODC is then 0.45 nmol per minute per mg of protein. A value of 1.94×10^8cells per mg protein [5] is applied first to count the number of cells per milligram of protein, resulting in the unit of μmol per minute per number of cells, which is further divided by total intracellular volume according to the previously stated relations between the number of cells and the volume value. A number of 40 μM/min is obtained for the maximum velocity of ODC (V_{max}^{ODC}). The same procedure is applied to converting the maximum velocity of SpdS given as 0.0119±0.0014 μmol per minute per mg of protein [6] into the unit of μM/min. A number of 1.0576e+03 μM/min is then obtained for the maximum velocity of SpdS (V_{max}^{SpdS}).

References

1. Hasne MP, Barrett MP (2000) Transport of methionine in *Trypanosoma brucei brucei*. Molecular and Biochemical Parasitology 111: 299–307.

2. Vincent IM, Creek DJ, Burgess K, Woods DJ, Burchmore RJS, et al. (2012) Untargeted metabolomics reveals a lack of synergy between nifurtimox and eflornithine against Trypanosoma brucei. PLoS Negl Trop Dis 6.

3. Opperdoes FR, Baudhuin P, Coppens I, Roe CD, Edwards SW, et al. (1984) Purification, morphometric analysis, and characterization of th glycosomes (microbodies) of the protozoan hemoflagellate *Trypanosoma brucei*. The Journal of Cell Biology 98: 1178–1184.

4. Phillips MA, Coffino P, Wang CC (1988) *Trypanosoma brucei* ornithine decarboxylase: enzyme purification, characterization, and expression in *Escherichia coli*. The Journal of Biological Chemistry 263: 17933–17941.

5. Bakker BM, Walsh MC, Kuile BHT, Mensonides FIC, Paul A M Michels FRO, et al. (1999) Contribution of glucose transport to the control of the glycolytic flux in trypanosoma brucei. PNAS 96: 10098–10103.

6. Taylor MC, Kaur H, Blessington B, Kelly JM, Wilkinson SR (2008) Validation of spermdine synthase as a drug target in african trypanosomes. Biochemical Journal 409: 563–569.
