# Supplementary material for: Mathematical Modelling of Polyamine Metabolism in Bloodstream-Form Trypanosoma brucei: An Application to Drug Target Identification
Source: PLoS One. 2013 Jan 23;8(1):e53734. doi: 10.1371/journal.pone.0053734 (PMC3553166; doi:10.1371/journal.pone.0053734)
Supplement: Text S3 — Parameter Estimation and Selection. Design of objective functions and selection of the best set of estimates. (PDF) [file pone.0053734.s007.pdf]

Supplementary text 3: Parameter Estimation and Selection

Direct determination of *in vivo* parameters is difficult and often noisy. Given sufficient experimental measurements of system components, the goal is to minimize the objective function that measures the quality of an estimated parameter set in satisfying a given criterion. In our study, unknown parameter values are refined by comparing simulation results with experimental measurements. A modified *G-test* was used as the objective function to measure the goodness of fit of the model defined in both conditions – steady-state condition and perturbed condition (DFMO-mediated inhibition). The general formula for our test statistic G is:

$$G(\mathbf{p}, t) = \sum_{t} \ln\left(\frac{O(t)}{E(\mathbf{p}, t)}\right) \tag{S1}$$

where **ln** denotes the natural logarithm and the sum is taken over all sampling points. In the context of optimization of biological pathways, there are discrete time points. Here, $\mathbf{p}$ is the decision vector to be tuned. $O(t)$ and $E(\mathbf{p}, t)$ are the observed data and model simulation, respectively, given at time point t.

This objective function (Equation S1) is sufficient to enable the acquisition of the perturbed condition of the model, which is constrained by the minimization of residuals. Under the steady-state condition, the concentration of model variables are constrained by two criteria: 1) the residual of observed data and simulation results and 2) the trend of change of concentrations. A feasible set of solutions should be tuned for the acquisition of both criteria. We use a dynamic penalty function to tackle the second criterion - the trend of change of concentrations. A general form of distance based penalty method, which incorporates a dynamic aspect based on length of search, l, is as follows, according to the description given by Smith and Coit [1]

$$f_p(\mathbf{x}, l) = f(\mathbf{x}) + \sum_{i=1} S_i(l) d_i^k \tag{S2}$$

where $S_i(l)$ is a function monotonically non-decreasing in value with l. Parameter d_i is the distance metric of constraint i (e.g. number of generations or the number of solutions searched) applied to solution set $\mathbf{x}$ and k is a user-defined exponent, with values of k of 1 or 2 often used.

The penalty function proposed in this study is defined as follow:

$$P(\mathbf{p}) = \sum_{n=1}^{m} n \cdot \ln\left(1 + \mid \frac{E(\mathbf{p}, n) - E(\mathbf{p}, n+1)}{E(\mathbf{p}, n)} \mid\right) \tag{S3}$$

where $\ln(1 + |\cdot|)$ is designed to accurately compute (small) absolute values in $|\cdot|$ that quantify the change in concentration of simulation results calculated at the boundaries of the time interval. Parameter n is the index of the time interval for $n = 1$ to m intervals in a time span divided into discrete time points and n is monotonically non-decreasing, acting as $S_i(l)$ in the general expression (Equation S2).

The idea of introducing the penalty function is to incorporate a dynamic aspect, which increases the severity of the penalty as the integration or simulation progresses. The objective function used for the acquisition of the steady-state condition is defined as a summation of both criteria as below (for a minimization problem):

$$G_p(\mathbf{p}, t) = G(\mathbf{p}, t) + P(\mathbf{p}) \tag{S4}$$

where $G(\mathbf{p}, t)$ is the unpenalized objective function and $P(\mathbf{p})$ is the penalty function.

In practice, penalty functions typically require problem specific tuning to perform well. A major difficulty of combining all objectives into a single objective lies in determining a multi-criteria fitness value to summarize the degree of satisfaction of all the individual objective. We investigated the appropriateness

of several definitions of the penalty function $P(\mathbf{p})$ differing in the explicit form of $S_i(l)$. The penalty function defined in Equation S3 was found to be the most suitable expression for the problem of interest.

In our study, five optimization runs (a maximum of 1200 iterations each) with random starting values of the unknown parameters are performed with MoPSwarm developed in [2] and multiple sets of estimates of unknown parameters were returned from the optimisation procedure (with the objective functions as defined above). MoPSwarm was developed on the basis of a Matlab implementation of the PSwarm algorithm [3], which aims to solve single-objective optimization problems via standard Particle Swarm optimization technique. PSwarm is designed around a Search and Poll framework, which has been demonstrated to outperform several global optimization methods, giving a better convergence rate and an optimal set of parameter estimations [3]. The original PSwarm Matlab code is available from the website http://www.norg.uminho.pt/aivaz/pswarm/.

The selection of the best trade-off solution is conducted according to a quantitative comparison method. This type of comparison method is based on a goodness-of-fit criterion, which is generally applied in the optimization problems where the emphasis is on the accuracy of the results. We employed the Root Mean Square (also known as the quadratic mean) to rank the solutions from all five simulations. This measure is a common method of defining the importance of solutions with respect to satisfying both steady-state and perturbed conditions, and is given as

$$\sqrt{\left(\frac{\mathrm{Obj}^i_{ss}}{\max(\mathrm{Obj}^i_{ss})}\right)^2 + \left(\frac{\mathrm{Obj}^i_{ptb}}{\max(\mathrm{Obj}^i_{ptb})}\right)^2} \tag{S5}$$

Here, Obj_{ss} and Obj_{ptb} stand for the objective functions of steady state and perturbed state of the pathway, respectively. Note that the objective function values have been normalized so that they fall in the range of 0 to 1, which prevents large absolute values from skewing the metric and gives equal weighting to both objectives.

References

1. Smith AE, Coit DW (1997) Handbook of Evolutionary Computation, Joint Publication of Oxford University Press and Institute of Physics Publishing, chapter Section C5.2, Penalty Functions.

2. Gu X (2010) Systems Biology Approaches to the Computational Modelling of Trypanothione Metabolism in Bloodstream-form *Trypanosoma brucei*. Ph.D. thesis, The University of Glasgow, Glasgow, UK.

3. Vaz AIF, Vicente LN (2007) A particle swarm pattern search method for bound constrained global optimization. Journal of Global Optimization 39: 197-219.
