# Supplementary material for: Mathematical Modelling of Polyamine Metabolism in Bloodstream-Form Trypanosoma brucei: An Application to Drug Target Identification
Source: PLoS One. 2013 Jan 23;8(1):e53734. doi: 10.1371/journal.pone.0053734 (PMC3553166; doi:10.1371/journal.pone.0053734)
Supplement: Text S4 — Analysis on the Model Refinements. Impact of the reversibility of enzyme ODC and the regulatory effect of SpdS on model behaviours. (PDF) [file pone.0053734.s008.pdf]

Supplementary text 4: Analysis on the Model Refinements

An iterative and incremental cycle between the steps of model construction and validation is often expected to render a model satisfactory. We substantiated this concept during the model building activities for the polyamine metabolic pathway in *T. brucei.* This means that deficiencies of the intermediate variants of the model could be resolved, leading to the proposal of two refinements (refer to Model V4 and V5 in Supplementary Table S2). Below, we illustrate the usefulness of these refinements in improving the capability of the model in representing reality, compared with the variants without these refinements.

Part 1. Modelling the ODC-catalyzed reaction reversibly

One of the refinements we made during the model building procedure was to represent the ODC-catalyzed reaction with the reversible rate law, as defined in Equation 1 in the section of Materials and Methods in the main text (also given below).

$$V_{ODC} = \left([Orn] - \frac{[Put]}{K_{eq}^{ODC}}\right) \cdot \left(\frac{\frac{V_{max}^{ODC} \cdot e^{\left(-\lambda_{SpdS} \cdot t\right)}}{K_{mOrn}^{ODC}}}{1 + \frac{[Orn]}{K_{mOrn}^{ODC}} + \frac{[Put]}{K_{mPut}^{ODC} \cdot (1 + \frac{[Put]}{K_{iP}^{ODC}})}} \right) \tag{ST1}$$

In this final model (Model V5 in Supplementary Table S2), we used the best set of parameter estimates obtained from solving Model V4, based on which we analytically derived the newly-added parameters - the equilibrium constant K_{eq}^{ODC} and the half-saturation constant K_{mPut}^{ODC}. Due to the lack of information on these two parameters, we assumed the half-saturation constant K_{mPut}^{ODC} to hold the same value as the known parameter K_{mOrn}^{ODC} and we analytically derived the equilibrium constant K_{eq}^{ODC} against the experimental observations of AdoMetDC RNAi induction and prozyme knockout.

We illustrate in Figure ST1 the effect of different values of parameter K_{eq}^{ODC} on the dynamic behaviour of Put in the cases of AdoMetDC RNAi induction and prozyme knockdown. This figure shows that our choice of K_{eq}^{ODC} (which was assigned the value of 5) best reflects the dynamics and exact concentration values of Put under the perturbed conditions and when compared with simulation results from modelling ODC irreversibly, Put dynamics could reach steady state gradually over time. When AdoMetDC or prozyme were inhibited, direct downstream metabolites of the ODC-catalyzed reaction - Spd and TSH_{tot}, were not different from the model simulations where ODC was described with the irreversible rate law, in terms of both the *basal* condition and dynamic behaviours (see Figure ST2), which is also the case under all other perturbed conditions considered in the manuscript. The similar effect on model behaviours exerted by varying K_{eq}^{ODC} was also seen on parameter K_{mPut}^{ODC} (varied between 0.1 to 100 fold of the assumed value, 350 μM). We also found that there is no obvious difference in model behaviour when the term representing production inhibition by Put is present or not, which is most likely due to the weak inhibitory effect exerted by Put.

Part 2. The postulated regulatory correlation of enzyme SpdS on ODC

We postulated in the main text that the regulation of enzyme SpdS on ODC is helpful for avoiding Put accumulation.

The regulation of SpdS on ODC is reflected by the term $e^{\left(-\lambda_{SpdS} \cdot t\right)}$ as defined in Equation ST1. In the model where the regulatory effect is not considered, parameter λ_{SpdS} is set to zero and the maximum velocity of ODC becomes time-invariant, whereas the model having this effect enabled allows the maximum velocity of ODC to vary with respect to changes in SpdS activity (in this case a value of 0.0016 is assigned to parameter λ_{SpdS} as used in the main text).

We examined this postulation by comparing Put dynamics (over a simulated time span of 6 days) with ODC modelled reversibly against the situation where ODC is modelled irreversibly, which is further compared in the presence and absence of the postulated regulation of SpdS on ODC. Figure ST3 shows that, in the absence of the postulated regulation, the reversible ODC kinetics helped bring down Put level in response to SpdS knockdown compared with the result from the irreversible kinetics; however, the resulting concentration of Put still increased nearly 20 fold of the control level. These comparison results imply that the postulated regulation of SpdS on ODC is still necessary in preventing Put accumulation regardless and the conclusion remains the same with different values of the equilibrium constant.

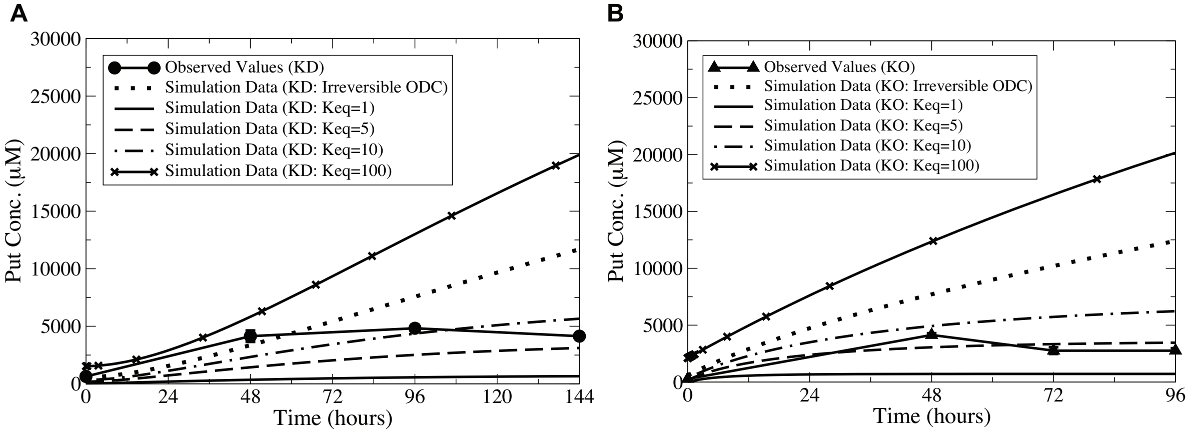

Figure ST1. Comparisons of Put dynamics in response to AdoMetDC RNAi induction (KD) and prozyme knockout (KO) when the ODC-catalyzed reaction was modelled with the irreversible or reversible (with different K_{eq}) rate law.

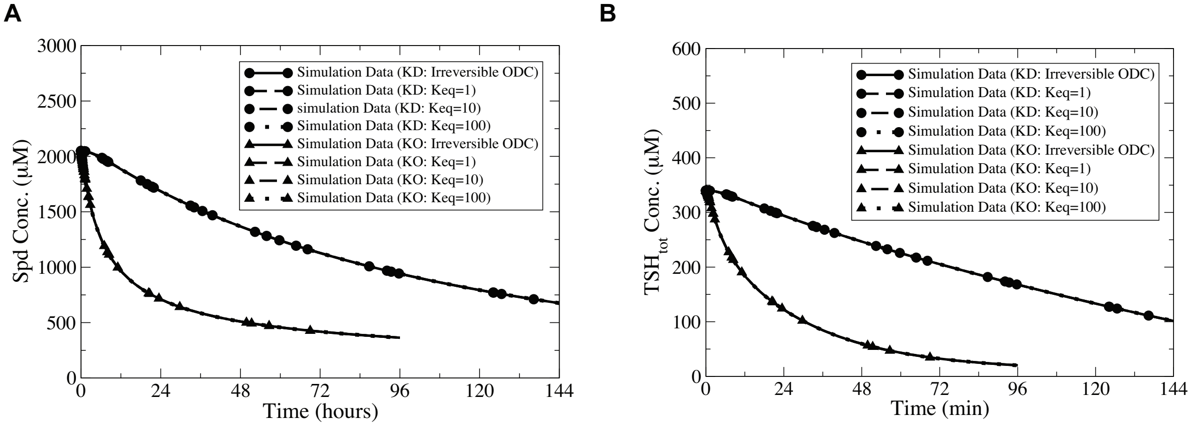

Figure ST2. Comparisons of Spd and TSH_{tot} dynamics in response to AdoMetDC RNAi induction (KD) and prozyme knockout (KO) when the ODC-catalyzed reaction was modelled with the irreversible or reversible (with different K_{eq}) rate law.

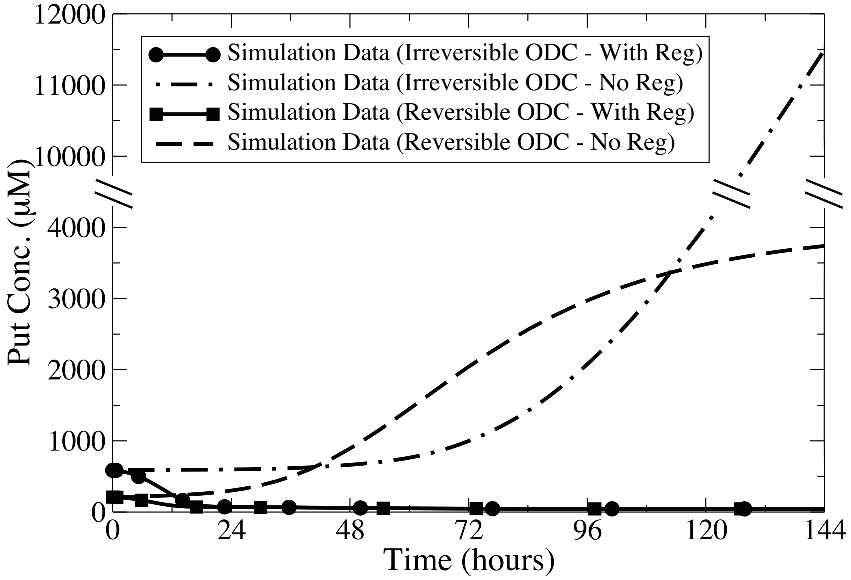

Figure ST3. Comparison of Put dynamics with and without the regulatory effect (Reg) of SpdS on ODC, when the kinetics of ODC is modelled reversibly and irreversibly, respectively.
